# Comparison of the efficacy and safety of first-line treatments for of advanced EGFR mutation-positive non-small-cell lung cancer in Asian populations: a systematic review and network meta-analysis

**DOI:** 10.3389/fphar.2023.1212313

**Published:** 2023-07-06

**Authors:** Wei Chen, Julian Miao, Ying Wang, Wenzhong Xing, Xiumei Xu, Rui Wu

**Affiliations:** ^1^ College of Pharmacy, Dali University, Dali, China; ^2^ The First People’s Hospital of Anning, Kunming, China

**Keywords:** EGFR-TKI, first-line treatment, NSCLC, bayesian network meta-analysis, bevacizumab, ramucirumab

## Abstract

**Background:** According to the 2023 guidelines for treating non-small-cell lung cancer (NSCLC), first-line treatment and recently developed agents for the treatment of epidermal growth factor (EGFR) mutation-positive locally advanced or metastatic NSCLC were compared in this meta-analysis. Treatment regimens involved in the included studies included first, second, and third-generation tyrosine kinase inhibitors (TKIs), TKIs plus chemotherapy, TKIs plus angiogenesis inhibitors, and platinum-containing doublet chemotherapy with or without bevacizumab. Considering the varying efficacy and safety of drugs in people of different ethnic origins, the optimal regimen should be determined, and the safety of first-line treatments should be assessed in the Asian population specifically.

**Methods:** PubMed, Embase, the Cochrane Library, Web of Science, and the China National Knowledge Infrastructure (CNKI) were systematically searched to retrieve reports on randomized controlled trials (RCTs) with research data published from inception to 1 February 2023. Adopting Asian patient populations as the target (including studies in which Asian patients accounted for more than 50% of the sample), a network meta-analysis (NMA) was conducted for comparison of treatment regimens and treatments were ranked based on the surface under the cumulative ranking curve (SUCRA).

**Results:** A total of 19 RCTs involving 5,824 patients and covering 14 treatment regimens were included. The primary outcome measure examined in this study was progression-free survival (PFS); other outcome measures examined were overall survival (OS), disease control rate (DCR), objective response rate (ORR), occurrence of any adverse events (AE), occurrence of adverse events of grade 3 or above (≥3AE), and occurrence of serious adverse events (SAE). In terms of PFS, all regimens including TKIs (as a monotherapy or in combination with other therapies), as well as bevacizumab (Bev) plus chemotherapy (Ch) were found to be significantly superior to basic chemotherapy (HRs: 0.09–0.61, *p* < 0.05 in all cases compared with Ch alone). The highest-ranking therapies were erlotinib (Erl) plus Bev (SUCRA: 0.94) and Erl plus ramucirumab (Ram) (SUCRA: 0.93). Regarding OS, no significant differences was observed between first-line treatment strategies; the top four treatments based on SUCRA, in rank order, were Bev + Ch (0.87), gefitinib (Gef) plus Ch (0.81), dacomitinib (Dac) (0.79), and osimertinib (Osi) (0.69). Additionally, there were no significant differences between first-line treatment strategies in terms of DCR. Regarding ORR, the top three treatments based on SUCRA were Erl + Bev (0.85), Erl + Ram (0.76), and Gef + Ch (0.74). No significant difference between first-line treatment strategies was observed in terms of the risk of AE. However, based on SUCRA, Erl ranked highest on avoidance of ≥ 3AE (0.97), and Osi ranked highest on avoidance of SAE (0.91).

**Conclusion:** Based on these analyses of survival benefits, tumor burden response, and safety, furmonertinib (Fur), Osi, and aumolertinib (Aum) may represent the best treatment regimen options for Asian patients, significantly prolonging survival (as measured by median PFS/OS), eliciting a greater tumor burden response, and exposing patients to a lower risk of adverse events. Although Erl + Bev and Erl + Ram are associated with the best survival benefits in terms of PFS, further clinical studies are still needed to identify ways to reduce the risk of adverse events.

**Systematic Review Registration:** https://www.crd.york.ac.uk/prospero/display_record.php? ID=CRD42023407994, identifier CRD42023407994

## 1 Introduction

Lung cancer is the most common malignant tumor appearing in clinical practice, ranking highest in terms of both incidence and mortality worldwide. According to global cancer statistics, there were more than 2.2 million new cases of lung cancer and more than 1.79 million deaths in 2020 (Sung et al., 2021), and non-small-cell lung cancer (NSCLC) accounted for approximately 85% of all lung cancer. The early stage of lung cancer can be cured by surgery combined with adjuvant treatment, but most patients are at an advanced stage when diagnosed (Chen et al., 2016). Previously, the median overall survival of patients with locally advanced or advanced lung cancer treated with chemotherapy was only approximately 1 year (Hu et al., 2019). In 2010, gefitinib (Gef) was examined as the first-line treatment for locally advanced or metastatic NSCLC patients with epidermal growth factor (EGFR)-sensitive mutations, achieving a median progression-free survival (mPFS) of 14 months and a median overall survival (mOS) of 30 months (Morikawa et al., 2015). The US FDA approved the third-generation EGFR TKI osimertinib (Osi) in 2018 as a first-line treatment for patients suffering from advanced or metastatic NSCLC harboring EGFR mutations (Soria et al., 2018; U.S.FDA., 2018); this treatment has been found to be associated with an mPFS of 19 months (Soria et al., 2018). Beyond these options, targeted drugs are widely used to treat advanced NSCLC with outstanding efficacy, good tolerance, and mild toxicity (Herbst et al., 2018). The primary driving genes in NSCLC include EGFR, KRAS, and ALK (Ettinger et al., 2017).

EGFR is a transmembrane receptor that activates the EGFR pathway, reduces autophagy in cancer cells, and promotes the secretion of growth factors in cancer cells, which is the main mechanism of tumor cell proliferation (Naylor et al., 2016; Itchins et al., 2018). Epidermal growth factor receptor tyrosine kinase inhibitors (EGFR TKIs) are an essential class of tumor-targeted therapeutics that are recommended as the standard first-line treatment for advanced NSCLC patients with EGFR mutations. Three generations of TKIs are currently available: for advanced NSCLC patients, first-generation TKIs can achieve an mPFS of more than 9 months (Maemondo et al., 2010), with the representative drugs being Gef and erlotinib (Erl), while second- and third-generation TKIs can achieve an mPFS of more than 19 months (Soria et al., 2018; Lu et al., 2022; Shi et al., 2022). Work by Nakagawa et al. (2019) indicates the efficacy of TKIs combined with anti-angiogenic agents in significantly prolonging PFS. Combination regimens, including Gef with chemotherapy (Ch) and Erl with bevacizumab (Bev) or ramucirumab (Ram), have been approved for first-line treatment, but multiple gene pathway treatments can overlap, leading to an increased risk of adverse events (Schulze et al., 2019).

There are differences between Asians and non-Asians in terms of driver gene mutations (Meng et al., 2019). The CTONG-0802 trial (Zhou et al., 2011) was conducted with an Asian population, and indicated an mPFS of 13.1 months for Erl (hazard ratio (HR) with chemotherapy: 0.16, 95% CI 0.10–0.26); in contrast, the EURTAC trial (Rosell et al., 2012) was conducted with a non-Asian population, and the results indicated an mPFS for Erl of only 9.7 months (HR: 0.37, 95% CI 0.25–0.54). The effect of the treatment was significantly better for Asian patients than for European and American Caucasian patients, and its efficacy varied between patients of different races. Differences between Eastern and Western populations in EGFR and KRAS mutations may also contribute to differences between different ethnic groups in terms of the benefits of targeted therapy. In the ARCHER 1050 trial (Wu et al., 2017), a between-group difference in hazard ratio for progression-free survival was observed for dacomitinib (Dac) in Asian vs non-Asian patients (Asian group, HR: 0.51, 95% CI 0.39–0.66; non-Asian group, HR: 0.89, 95% CI 0.57–1.39). Similarly, in the LUX-Lung3 trial (Sequist et al., 2013), a difference between the groups was observed in terms of hazard ratio for progression-free survival for afatinib (Afa) in Asian vs non-Asian patients (Asian group, HR: 0.54, 95% CI 0.38–0.76; non-Asian group, HR: 0.68, 95% CI 0.39–1.19). Finally, the results of the FLAURA trial (Ramalingam et al., 2020) suggested the largest between-group difference in relation to the hazard ratio for Osi in terms of overall survival in Asian vs non-Asian patients (Asian group, HR: 1.00, 95% CI 0.75–1.32; non-Asian group, HR: 0.54, 95% CI 0.38–0.77). Overall, these results suggest significant differences in treatment efficacy between people of different races. According to 2023 guidelines, the first-line treatment for advanced EGFR mutation-positive NSCLC is Osi (preferred), Gef, Erl, icotinib (Ico), Afa, Dac, aumolertinib (Aum), Gef/Erl + Ch, Erl + Ram, Erl + Bev, and Bev + Ch. Thus, many first-line treatment strategies are available, but no RCT has been conducted for direct comparison of their efficacy and safety, so the best option remains unclear.

In 2022, Chen et al. (2022) conducted a network meta-analysis (NMA) of first-line treatment strategies for patients with NSCLC, and found that second-generation TKIs, third-generation TKIs, and combination therapy were superior to first-generation TKIs in terms of PFS. However, there was no significant difference in OS between the different groups. Yang et al. (2022) also conducted an NMA; they found that Osi ranked first in terms of mPFS, and the combined scheme Gef plus pemetrexed-based chemotherapy (PB) was the most effective in terms of OS. However, the results varied, and subgroup analyses by race were not performed. Since these publications, many studies have provided updated data (Hosomi et al., 2022; Kawashima et al., 2022; Lu et al., 2022; Shi et al., 2022), and differences between study populations have been found to exert an impact on observed efficacy. Most existing RCTs have been conducted with Asian populations, making it difficult to perform analyses comparing efficacy for different races. In the present study, an NMA was conducted for studies of 14 first-line treatment regimens (Gef, Erl, Ico, Afa, Dac, Osi, Aum, furmonertinib (Fur), Erl + Ram, Erl + Bev, Gef + Ch, Ico + Ch, Bev + Ch, and Ch) in Asian patients in order to compare their effectiveness and safety and to explore the optimal first-line regimen for Asian patients.

## 2 Materials and methods

### 2.1 Literature search strategy

This study followed the reporting guidelines and extension statements of the Preferred Reporting Initiative for Systematic Reviews and Meta-analyses (PRISMA) (Hutton et al., 2015), and was registered with PROSPERO (CRD42023407994). Publications reporting on RCTs were collected from December 2022 onward by searching PubMed, Embase, the Cochrane Library, Web of Science, the China National Knowledge Infrastructure (CNKI), and the China Biomedical Literature Database (CBM) using keywords including “Gefitinib”, “Erlotinib”, “Icotinib”, “Afatinib”, “Dacomitinib”, “Osimertinib”, “Aumolertinib”, “Furmonertinib”, “Bevacizumab”, “Ramucirumab”, and “Non-Small Cell Lung Cancer”. The study type was limited to “randomized controlled trial (RCT)". Up to 01 February 2023, the language of publication was restricted to English or Chinese. A detailed description of the retrieval methods is provided in Supplementary Data Sheet S1. Additionally, the WHO International Clinical Trials Registry Platform (ICTRP), the Chinese Clinical Trials Registry, and ClinicalTrials.gov were searched for ongoing or unpublished data. To avoid missing articles through keyword searches, additional articles were also obtained by searching the citation lists of included articles and recent reviews. Two investigators independently assessed the articles for eligibility. Any disagreement was resolved through further discussion with a third investigator.

### 2.2 Selection criteria

The inclusion criteria were as follows: 1) study subjects: EGFR mutation-positive patients with locally advanced/advanced NSCLC; 2) study type: RCT; 3) interventions: trials where at least two groups of first-line treatments were compared, and at least one intervention was one of Gef, Erl, Ico, Afa, Dac, Osi, Aum, Fur, Erl + Ram, Erl + Bev, Gef/Erl + Ch, or Bev + Ch; and 4) at least one of the following outcomes was reported: objective response rate (ORR), disease control rate (DCR), PFS, OS, occurrence of any adverse event (AE), occurrence of any AE of grade 3 or above (≥3AE), and occurrence of any serious AE (SAE).

The exclusion criteria were as follows: 1) specific publication types without corresponding available data, such as letters, comments, editorials, agreements, replies, reviews, or guidelines; 2) insufficient data reported in the article; 3) < 50% of patients drawn from an Asian population; 4) retrospective case studies and case reports on a limited number of cases; and 5) upon full-text review, no data were available.

The most recently published, up-to-date data were adopted in the case of studies collected multiple times over time.

### 2.3 Data extraction

Data were extracted independently from each article by two of the authors, their quality was assessed, and they were recorded using Excel. The data extracted from the included articles were first author, publication year, country, number and characteristics of patients, interventions, and outcomes. The outcome indicators included tumor response (ORR and DCR), survival indicators (HR with 95% CI for PFS and OS), and safety indicators (AE, ≥3 AE, and SAE). The Cochrane Risk of Bias tool (Martimbianco et al., 2023) was used to assess the risk of bias for each study in areas including randomization, allocation concealment, blinding, completeness of outcomes, selective outcome reporting, and other sources of bias; quality assessment was performed using Revman 5.3. Two investigators independently conducted data extraction and risk of bias assessments, and discrepancies were resolved by comparison with the judgment of a third investigator.

### 2.4 Data analysis

Pooled data were entered into the analyses. The results were calculated using a Bayesian algorithm. Survival class data (PFS and OS) are reported in the form of HRs and corresponding 95% CIs to represent effect sizes. To guarantee the stability of the model, a random effects consistency model was used in the NMA. All statistical analyses were conducted using Stata 16.0 and R 4.2.0. For PFS and OS, analysis was carried out using the ‘GEMTC’ and ‘JAGS 4.3.0’ packages in R (version 4.2.0) (Shim et al., 2019) with 20,000 sample iterations, 5,000 burns, and a rarefaction interval of 1. The HR was used as a representation of the effect size, with a smaller HR indicating a larger effect. In addition, network consistency was evaluated via the node-splitting technique. A *p*-value less than 0.5 was taken to indicate significant inconsistency (van Valkenhoef et al., 2016), and trajectory plots took convergence into account. Dichotomized data on ORR, DCR, AE, ≥3AE, and SAE were analyzed in the form of ORs and corresponding 95% CIs; this analysis was conducted using the ‘netmeta’ package in Stata16. The OR was taken as a representation of the effect size. In the cases of DCR and ORR, the larger the OR value was, the better the tumor load response was; the opposite was the case for the outcome measures of AE, ≥3 AE, and SAE, where a larger value indicated a higher risk of adverse events. Heterogeneity was additionally assessed by calculating I^2^ for all studies. Following these analyses, all treatments were ranked according to the surface under the cumulative ranking curve (SUCRA) (Daly et al., 2019), where the higher the SUCRA score, the better the efficacy or safety of the treatment regimen. Finally, subgroup analyses were conducted using Revman 5.3.

## 3 Results

### 3.1 Search results and study characteristics

A total of 13,551 articles were preliminarily retrieved in this study. Duplicate articles (6,223 records) and articles reporting on irrelevant interventions were excluded. A comprehensive review of the remaining 28 articles was conducted (Cheng et al., 2016; Hosomi et al., 2020; Hosomi et al., 2022; Kawashima et al., 2022; Lu et al., 2022; Maemondo et al., 2010; Mitsudomi et al., 2010; Mok et al., 2021; Nakagawa et al., 2019; Noronha et al., 2020; Park et al., 2016; Paz-Ares et al., 2017; Ramalingam et al., 2020; Saito et al., 2019; Sequist et al., 2013; Seto et al., 2014; Shi et al., 2017; Shi et al., 2022; Soria et al., 2018; Wu et al., 2014; Wu et al., 2017; Xu et al., 2019; Yang et al., 2015; Yang et al., 2020; Yoshioka et al., 2019; Zhou et al., 2011; Zhou et al., 2015 1); Zhou et al., 2015 2)), and these were found to include 19 reports on RCTs (see Table 1 for details).

**TABLE 1:** RCTs included in the meta-analysis.

Study		Country and participant population	Treatment strategies	Sample size	Outcome measures
APOLLO NCT03849768 III	Lu et al. (2022)	China; 100% Asian	Aum (110 mg aumolertinib)	214	PFS, DCR, ORR, AE, ≥3AE, SAE
			Gef (250 mg gefitinib)	215	
FLAURA NCT02296125 III	Soria et al. (2018)	International; 62% Asian	Osi (80 mg osimertinib)	279	OS, PFS, DCR, ORR, AE, ≥3AE, SAE
	Ramalingam et al. (2020)		Gef (183: 250 mg gefitinib); Erb (94: 150 mg erlotinib)	277	
FURLONG NCT03787992 III	Shi et al. (2022)	China; 100% Asian	Fur (80 mg furmonertinib)	178	PFS, DCR, ORR, AE, ≥3AE, SAE
			Gef (250 mg gefitinib)	179	
LUX-Lung7 NCT01466660 IIB	Park et al. (2016)	International; 57% Asian	Afa (40 mg Afatinib)	160	OS, PFS, DCR, ORR, AE, ≥3AE, SAE
	Paz-Ares et al. (2017)		Gef (250 mg Gefitinib)	159	
LUX-Lung3 III	Sequist et al. (2013)	International; 72% Asian	Afa (40 mg afatinib)	230	OS, PFS, DCR, ORR, ≥3AE
	Yang et al. (2015)		Ch (cisplatin plus pemetrexed chemotherapy)	115	
LUX-Lung6 NCT01121393 III	Wu et al. (2014)	International; 100% Asian	Afa (40 mg afatinib)	242	OS, PFS, DCR, ORR, AE, ≥3AE, SAE
	Yang et al. (2015)		Ch (gemcitabine plus cisplatin)	122	
CONVINCE III	Shi et al. (2017)	China; 100% Asian	Ico (125 mg icotinib)	148	OS, PFS, AE, ≥3AE
			Ch (cisplatin plus pemetrexed)	137	
NCT02031601 III	Xu et al. (2019)	China; 100% Asian	Ico + Ch (pemetrexed and carboplatin plus icotinib)	90	OS, PFS, DCR, ORR
			Ico (125 mg icotinib)	89	
ARCHER 1050 NCT01774721 III	Wu et al. (2017)	International; 77% Asian	Dac (45 mg dacomitinib)	227	OS, PFS, DCR, ORR, AE, ≥3AE, SAE
	Mok et al. (2021)		Gef (250 mg gefitinib)	225	
NCT02411448 III	Nakagawa et al. (2019)	International; 77% Asian	Erl (erlotinib 150 mg/day)	225	PFS, DCR, ORR, AE, ≥3AE, SAE
			Erl + Ram (ramucirumab 10 mg/kg plus erlotinib 150 mg/day)	224	
UMIN000017069 III	Saito et al. (2019)	Japan; 100% Asian	Erl (150 mg erlotinib)	112	OS, PFS, DCR, ORR, AE, ≥3AE
	Kawashima et al. (2022)		Erl + Bev (erlotinib 150 mg plus bevacizumab 15 mg/kg)	112	
JO25567 JapicCTI-111390 II	Seto et al. (2014)	Japan; 100% Asian	Erl (150 mg erlotinib)	77	OS, PFS, DCR, ORR, AE, ≥3AE, SAE
	Hosomi et al. (2022)		Erl + Bev (erlotinib 150 mg plus bevacizumab 15 mg/kg)	75	
CTONG-0802 NCT00874419 III	Zhou et al. (2011)	China; 100% Asian	Erl (150 mg erlotinib)	82	OS, PFS, DCR, ORR, AE, ≥3AE, SAE
	Zhou et al. (2015a)		Ch (gemcitabine plus carboplatin)	72	
C000000376 III	Maemondo et al. (2010)	Japan; 100% Asian	Gef (250 mg gefitinib)	114	OS, PFS, DCR, ORR, AE, ≥3AE
			Ch (paclitaxel plus carboplatin)	114	
WJTOG3405 III	Mitsudomi et al. (2010)	Japan; 100% Asian	Gef (250 mg gefitinib)	86	OS, PFS, DCR, ORR
	Yoshioka et al. (2019)		Ch (docetaxel plus cisplatin)	86	
NCT01469000 II	Cheng et al. (2016)	International; 100% Asian	Gef (250 mg gefitinib)	65	OS, PFS, DCR, ORR, AE, ≥3AE, SAE
	Yang et al. (2020)		Gef + Ch (pemetrexed plus gefitinib 250 mg)	126	
CTRI/2016/08/007,149 III	Noronha et al. (2020)	India; 100% Asian	Gef (250 mg gefitinib)	176	PFS, DCR, ORR, ≥3AE, SAE
			Gef + Ch (gefitinib 250 mg plus pemetrexed and carboplatin)	174	
NEJ009 III	Hosomi et al. (2020)	Japan; 100% Asian	Gef (250 mg gefitinib)	172	OS, PFS, DCR, ORR, AE, ≥3AE
			Gef + Ch (gefitinib 250 mg plus carboplatin and pemetrexed)	170	
BEYOND III	Zhou et al. (2015b)	China; 100% Asian	Bev + Ch (carboplatin and paclitaxel plus bevacizumab 15 mg/kg)	138	OS, PFS, DCR, ORR, ≥3AE, SAE
			Ch (carboplatin and paclitaxel)	138	

PFS, progression-free survival; OS, overall survival; DCR, disease control rate; ORR, objective response rate; AE, occurrence of any adverse events; ≥3 AE, occurrence of any adverse events of grade 3 or above; SAE, occurrence of any serious adverse events.

**FIGURE 1:**
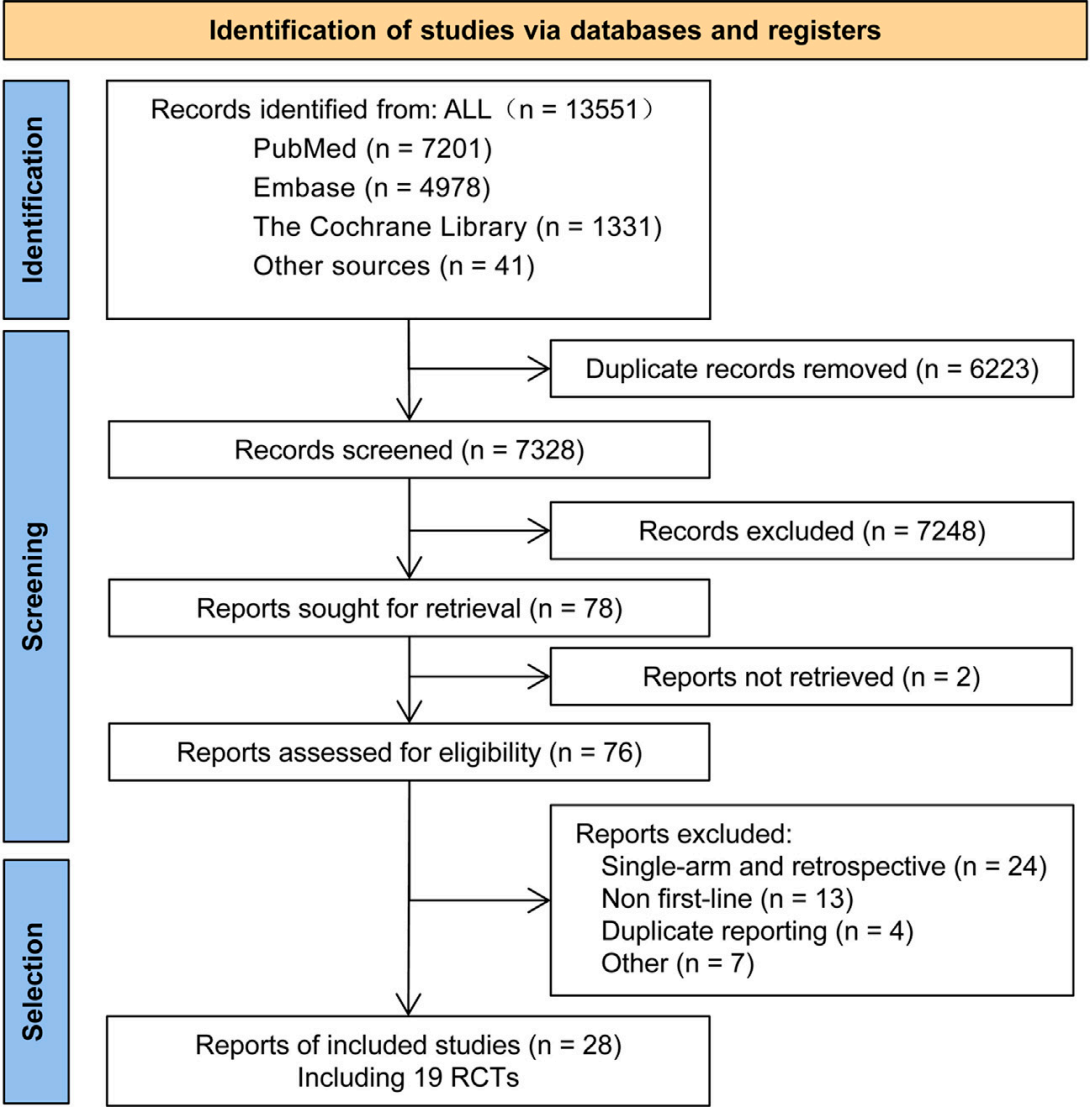
Flow chart showing the literature screening and selection process.

 A flow chart illustrating the process of research literature retrieval and screening is shown in Figure 1. The included studies examined 14 types of treatment regimen, which fell into three categories: TKI monotherapies (Gef, Erl, Ico, Afa, Dac, Aum, Osi, and Fur), TKIs combined with anti-angiogenic agents (Erl + Bev and Erl + Ram), and chemotherapy-related treatment (Gef + Ch, Ico + Ch, Bev + Ch, and Ch only).

The 19 included studies were mainly international multicenter RCTs, involving a total of 5,824 patients. The patients were recruited mainly from Asian populations; the median age fell between 56.0 and 64.2 years; women accounted for approximately 65% of patients; non-smokers accounted for approximately 85%; and ECOG scores were mainly 0 or 1, with adenocarcinoma patients accounting for over 90% of patients (see Supplementary Table S1 for details).

### 3.2 Quality assessment, convergence, and heterogeneity analysis

**FIGURE 2:**
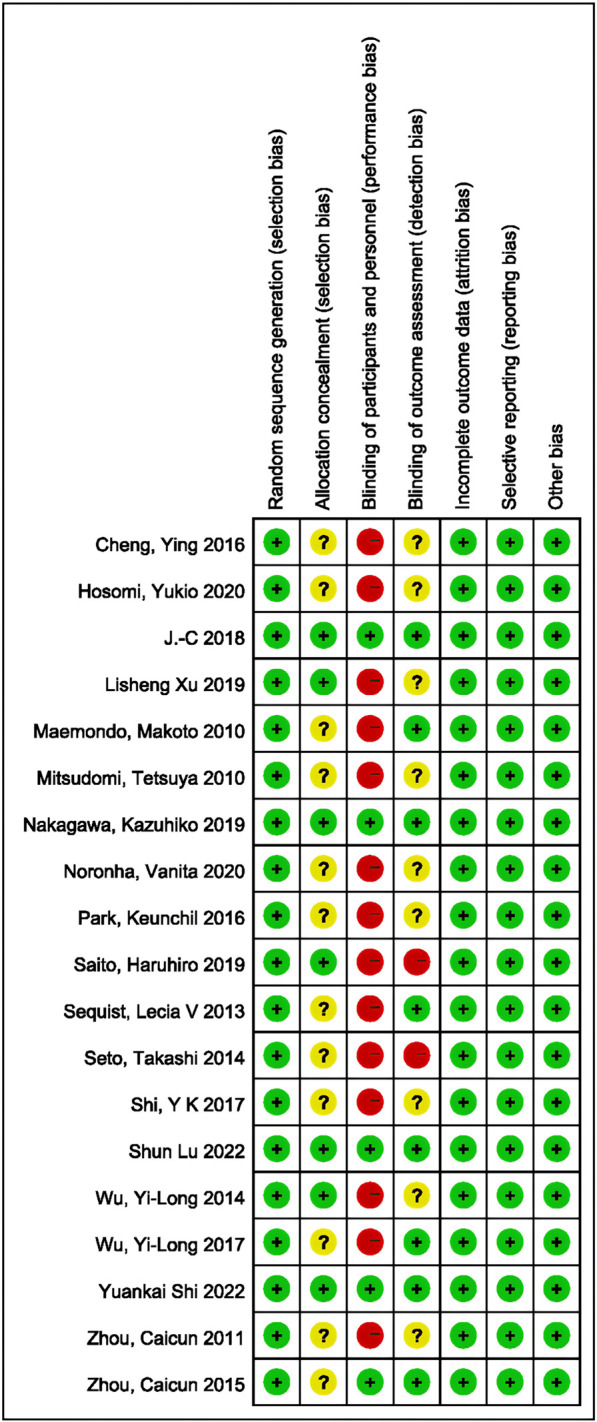
Summary of risk-of-bias analysis. Green represents a low risk of bias, yellow an unclear risk, and red a high risk.

The methodological quality of the included trials was generally high according to the Cochrane risk-of-bias criteria. In all trials, random sequence generation was sufficient. The concealment of assignments was not reported in most trials. An open-label design was employed in 14 of the included studies, in which neither the investigator nor the patient was blinded to treatment allocation. Of these, 5 studies scored more than 6 points; see Figure 2 for details.

Convergence diagrams for PFS and OS are shown in Supplementary Data Sheet S2 (Supplementary Figures S1, S2, respectively). Funnel plots for all results (Supplementary Figures S2, S3) were almost symmetric, indicating no significant inconsistencies. The lack of a closed loop in the network diagram made it impossible to carry out a detailed inconsistency analysis. The contribution ratio of all results combined with direct and indirect comparisons is shown in Supplementary Data Sheet S2 (Supplementary Figure S4).

### 3.3 Efficacy: PFS and OS

**FIGURE 3:**
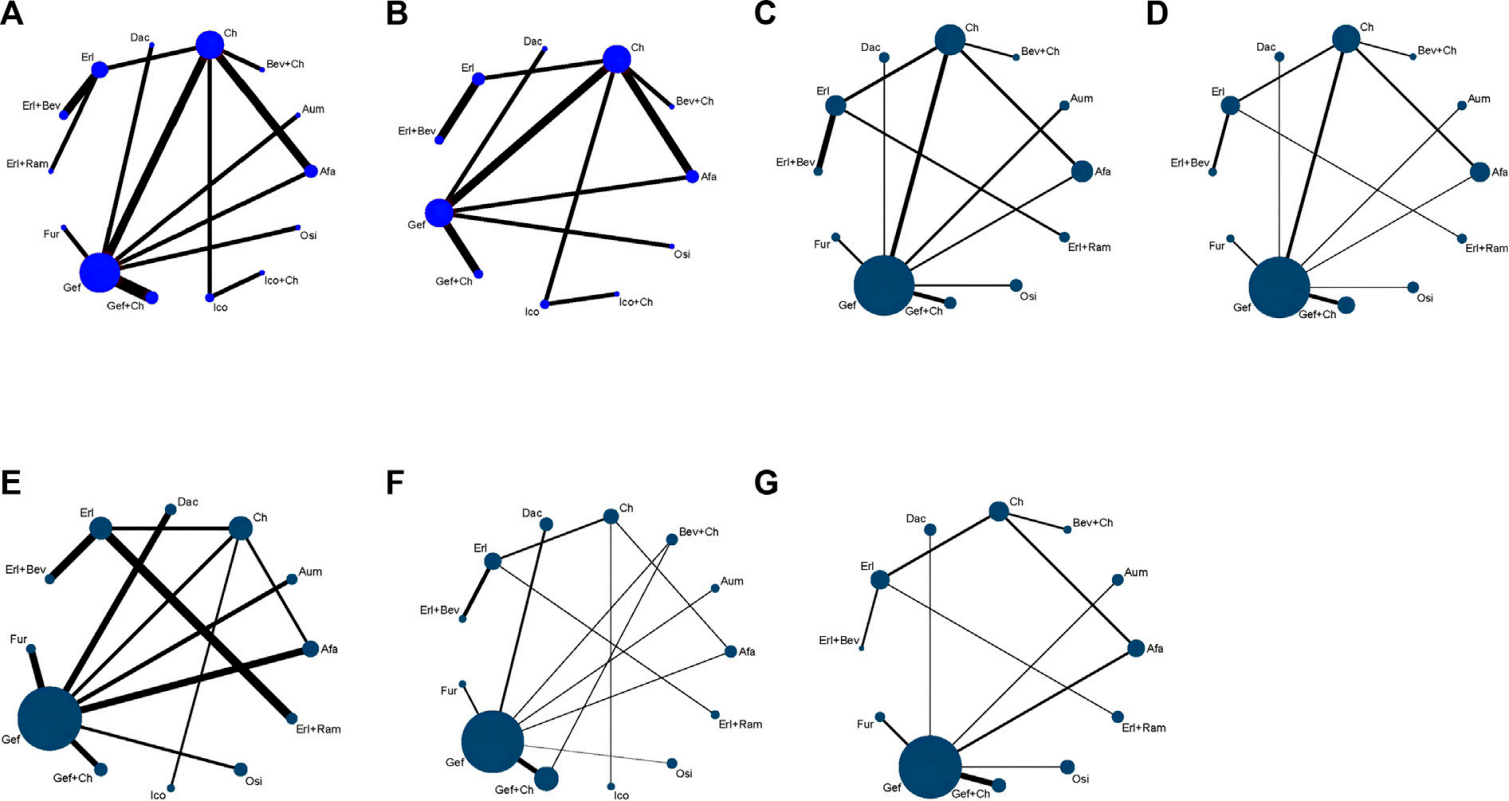
Network evidence plots showing outcomes of comparisons of treatments in patients with advanced EGFR-mutated NSCLC for **(A)** PFS **(B)** OS **(C)** DCR **(D)** ORR **(E)** risk of AE **(F)** risk of ≥3 AE, and **(G)** SAE. Each circular node represents a type of treatment. Each line represents a head-to-head comparison between two types of treatment. Panels **(A, B)** illustrate survival time data, with the size of the node and the thickness of the line weighted according to the number of studies evaluating each treatment and each direct comparison, respectively. Panels **(C–G)** present binary data, with the size of the node and the thickness of the line weighted according to the number of study subjects evaluated for each treatment and for each direct comparison, respectively. Afa, afatinib; Aum, aumolertinib; Bev, bevacizumab; Dac, dacomitinib; Erl, erlotinib; Fur, furmonertinib; Gef, gefitinib; Ico, icotinib; Ch, basic chemotherapy; Osi, osimertinib; Ram, ramucirumab.

Relevant data on PFS (19 studies) and OS (15 studies) were extracted from the selected RCTs; these outcome measures were assessed for 14 and 11 treatment options, respectively, and the corresponding network relationships are shown in Figures 3A,B.

**FIGURE 4:**
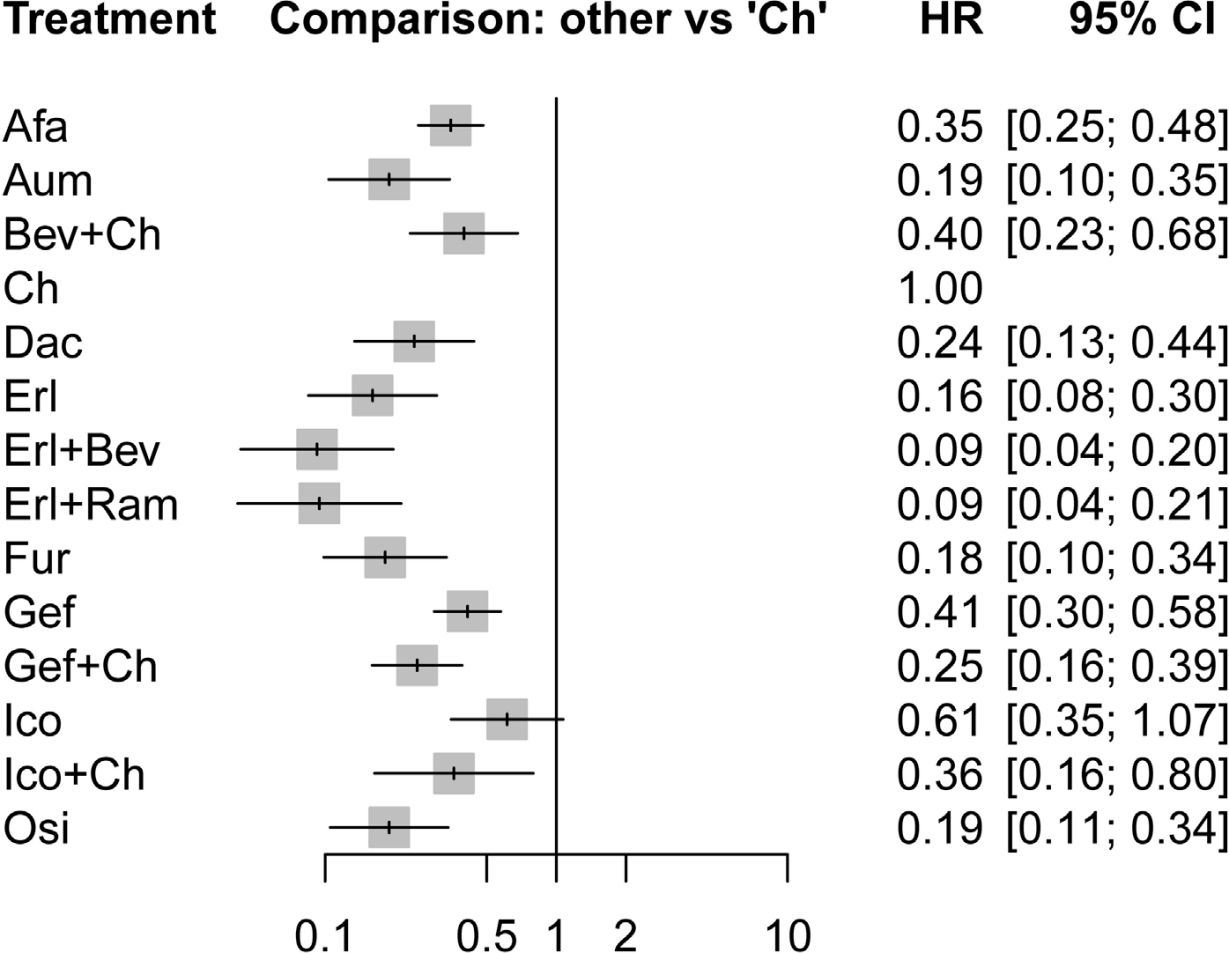
Forest plot comparing multiple interventions with Ch in terms of PFS. Afa, afatinib; Aum, aumolertinib; Bev, bevacizumab; Dac, dacomitinib; Erl, erlotinib; Fur, furmonertinib; Gef, gefitinib; Ico, icotinib; Ch, basic chemotherapy; Osi, osimertinib; Ram, ramucirumab; CI, confidence interval; HR, hazard ratio.

**FIGURE 5:**
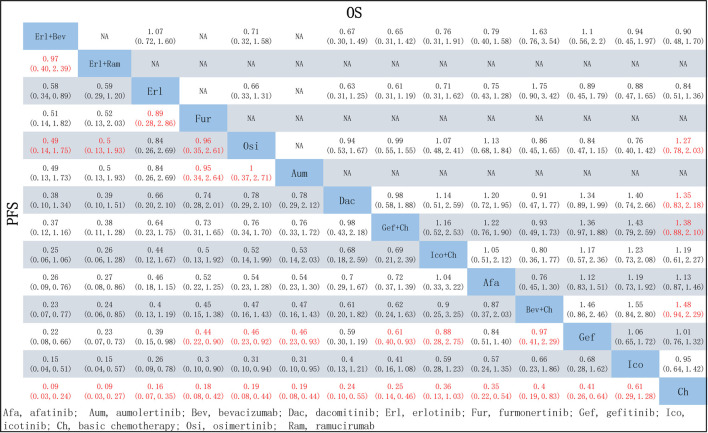
Pooled comparisons of treatments in patients with NSCLC with EGFR mutations, presented in the form of HR (95% CI) for PFS (lower triangle) and OS (upper triangle). Each cell represents a comparison between the treatment specified on that row and the one specified in that column. An HR < 1 favors the therapy defined in the column. Important results are highlighted in red font. PFS, progression-free survival; OS, overall survival.

In terms of PFS, all treatment regimens incorporating TKIs (single-drug or combination therapy), along with Bev + Ch, were significantly superior to Ch only (HR: 0.09–0.61, *p* < 0.05), as shown in Figure 4. Treatments consisting of a TKI combined with angiogenesis inhibitors were significantly superior to other treatment regimens: specifically, these regimens were superior to Osi (Erl + Bev: HR = 0.49, 95% CI: 0.14–1.75; Erl + Ram: HR = 0.5, 95% CI: 0.13–1.93), Gef (Erl + Bev: HR = 0.22, 95% CI: 0.08–0.66; Erl + Ram: HR = 0.23, 95% CI: 0.07–0.73), Gef + Ch (Erl + Bev: HR = 0.37, 95% CI: 0.12–1.16; Erl + Ram: HR = 0.38, 95% CI: 0.11–1.28), Afa (Erl + Bev: HR = 0.26, 95% CI: 0.09–0.760; Erl + Ram: HR = 0.27, 95% CI: 0.08–0.86). Additionally, compared to first-generation TKIs (Gef), third-generation TKIs showed significantly more beneficial effects: this was the case for Osi (HR = 0.46, 95% CI: 0.23–0.92), Aum (HR = 0.46, 95% CI: 0.23–0.93), and Fur (HR = 0.44, 95% CI: 0.22–0.90); but there was no statistically significant difference in the case of Erl. Third-generation TKIs were also significantly more beneficial than second-generation TKIs (Afa/Dac): specifically, in comparison to Afa, significantly better outcomes were observed for Osi (HR = 0.54, 95% CI: 0.23–1.28), Aum (HR = 0.54, 95% CI: 0.23–1.30), and Fur (HR = 0.52, 95% CI: 0.22–1.25). Compared with Gef, PFS was significantly prolonged by treatment with Ch in combination with other treatments: specifically, outcomes were significantly better for Gef + Ch (HR = 0.61, 95% CI: 0.40–0.93), Ico + Ch (HR = 0.88, 95% CI: 0.28–2.75), and Bev + Ch (HR = 0.97, 95% CI: 0.41–2.29). Other differences were not statistically significant. Additionally, there was no statistically significant difference between Erl + Ram and Erl + Bev, and there were no statistically significant differences between third-generation TKIs (see Figure 5 for details).

**FIGURE 6:**
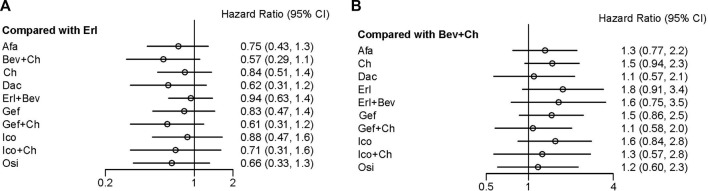
Forest plot comparing multiple interventions with **(A)** Erl and **(B)** Bev + Ch in terms of OS. Afa, afatinib; Aum, aumolertinib; Bev, bevacizumab; Dac, dacomitinib; Erl, erlotinib; Fur, furmonertinib; Gef, gefitinib; Ico, icotinib; Ch, basic chemotherapy; Osi, osimertinib; Ram, ramucirumab; CI, confidence interval.

In terms of OS, data were not mature in the cases of Aum, Fur, and Ico + Ch. Based on the data available, several treatment regiments were superior to chemotherapy only in improving OS: specifically, compared with Ch, more beneficial effects were found for Bev + Ch (HR = 0.68, 95% CI: 0.44–1.06), Gef + Ch (HR = 0.73, 95% CI: 0.48–1.14), Dac (HR = 0.74, 95% CI: 0.46–1.21), and Osi (HR = 0.79, 95% CI: 0.49–1.28). There were no other significant differences between groups in terms of first-line treatment strategies (details are provided in Figure 5). All other treatments showed more beneficial effects compared with Erl (HR < 1, *p* < 0.05 in all cases; see Figure 6A). Finally, the results also indicated that Bev + Ch had a more beneficial effect on OS than all other treatments (Figure 6B).

### 3.4 Tumor load response: DCR and ORR

**FIGURE 7:**
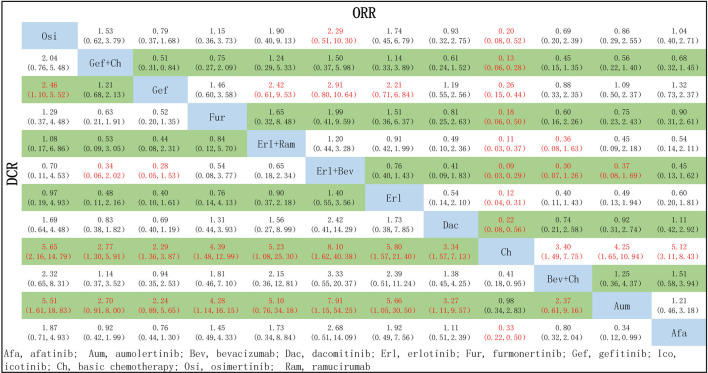
Pooled comparisons of treatments in patients with NSCLC with EGRF mutations, presented in the form of OR (95% CI) for DCR (lower triangle) and ORR (upper triangle). Each cell represents a comparison between the treatment specified on that row and the one specified in that column. An OR >1 favors the therapy defined in the column. Important results are highlighted in red font. DCR, disease control rate; ORR, objective response rate.

A total of 18 studies reported relevant data on tumor load response, and NMA was conducted for 17 studies (no network could be formed for the study by Xu et al. (2019), which therefore could not be analyzed), covering 12 treatment options. The network relationships are shown in Figures 3C,D. There was no statistically significant difference between the first-line treatment strategy groups in terms of DCR. In the case of ORR, third-generation TKIs were associated with better tumor load responses than first-generation TKIs: specifically, in comparison to Gef, superior effects were observed for Osi (OR = 1.27, 95% CI: 0.59–2.72), Aum (OR = 1.09, 95% CI: 0.50–2.37), and Fur (OR = 1.46, 95% CI: 0.60–3.68). Additionally, treatments with Erl combined with anti-angiogenic drugs were associated with improved ORR compared to single-drug TKI treatments: specifically, better tumor load responses were found for these treatments compared to Erl (Erl + Bev: OR = 1.32, 95% CI 0.70–2.48; Erl + Ram: OR = 1.09, 95% CI 0.50–2.38), Gef (Erl + Bev: OR = 2.91, 95% CI 0.80–10.64; Erl + Ram: OR = 2.42, 95% CI 0.61–9.53), Osi (Erl + Bev: OR = 1.90, 95% CI 0.40–9.13; Erl + Ram: OR = 2.29, 95% CI 0.51–10.30, and Afa (Erl + Bev: OR = 2.21, 95% CI 0.62–7.93; Erl + Ram: OR = 1.84, 95% CI 0.47–7.11). All other therapies showed better tumor load responses compared with Ch only (ORs: 3.40–11.33, *p* < 0.05 in all cases). Finally, Erl + Bev was associated with the best tumor load response (ORs relative to other therapies: 0.09–0.83, *p* < 0.05), as detailed in Figure 7.

### 3.5 Safety: AE, ≥3AE, and SAE

A total of 17 studies reported data relevant to safety; NMA was conducted for these, covering 13 treatment schemes. The network relationships are shown in Figures 3E–G.

**FIGURE 8:**
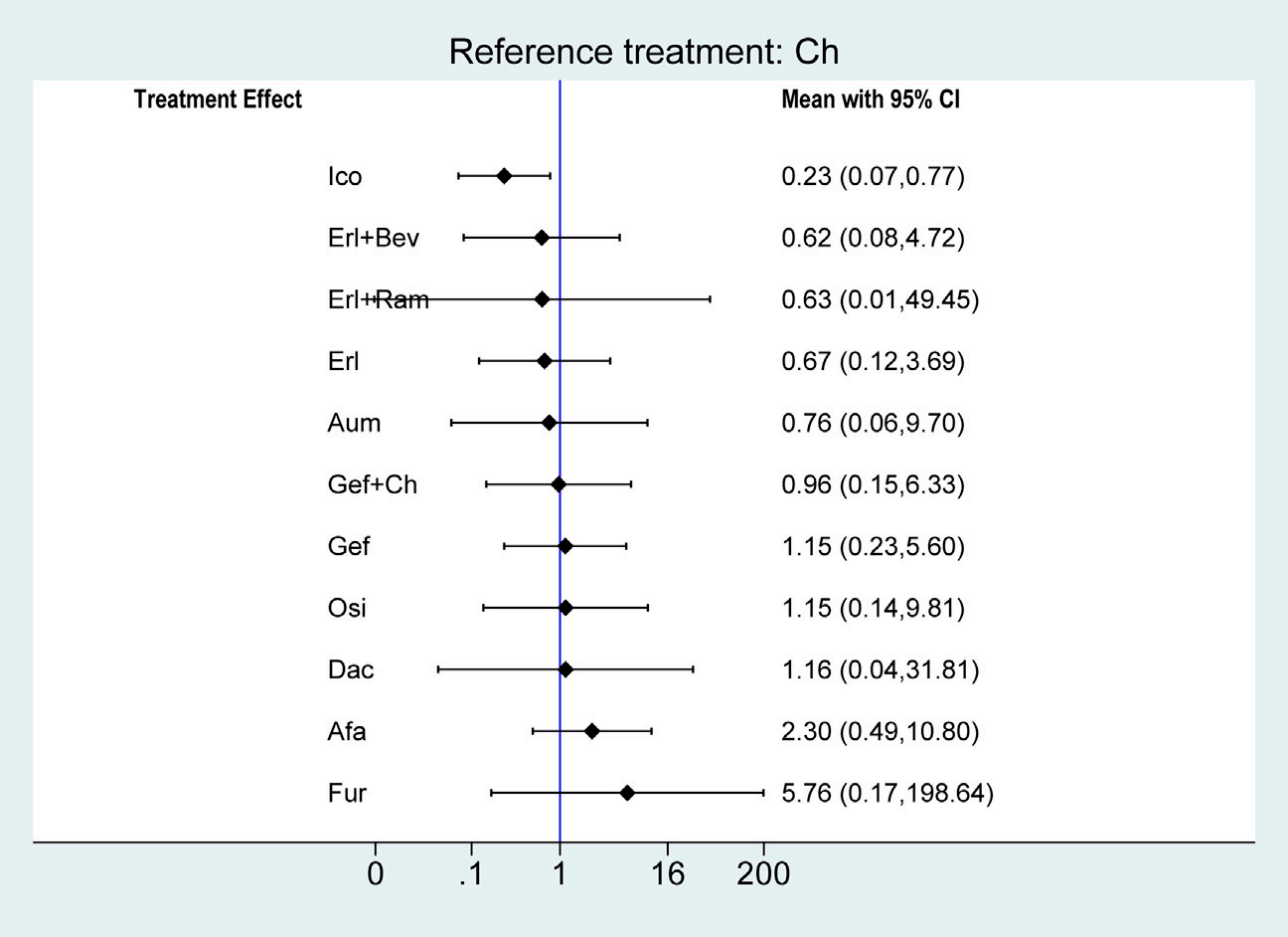
Forest plots comparing multiple interventions with Ch in terms of the incidence of any AE. Afa, afatinib; Aum, aumolertinib; Bev, bevacizumab; Dac, dacomitinib; Erl, erlotinib; Fur, furmonertinib; Gef, gefitinib; Ico, icotinib; Ch, basic chemotherapy; Osi, osimertinib; Ram, ramucirumab; CI, confidence interval.

Regarding AE, there were no statistically significant differences in treatment safety among the groups, except in the case of Ico, which significantly reduced the risk of an AE compared with chemotherapy (OR = 0.23, 95% CI: 0.07–0.77), as shown in Figure 8.

In terms of ≥3AE, most single-agent TKI treatments were associated with a significantly reduced risk of ≥3AE compared with chemotherapy: specifically, this was the case for Erl (OR = 0.11, 95% CI: 0.04–0.30), Fur (OR = 0.23, 95% CI: 0.07–0.68), Osi (OR = 0.24, 95% CI: 0.09–0.66), Gef (OR = 0.39, 95% CI: 0.20–0.74), Aum (OR = 0.40, 95% CI: 0.15–1.10), Afa (OR = 0.61, 95% CI: 0.34–1.02), and Ico (OR = 0.67, 95% CI: 0.33–1.75), but not for Dac (OR = 0.96, 95% CI: 0.35–2.61). Additionally, treatment with Erl combined with angiogenesis inhibitors resulted in a significantly higher risk of ≥3AE compared with Erl only: this was the case for both Erl + Ram (OR = 2.20, 95% CI: 1.02–4.78) and Erl + Bev (OR = 8.09, 95% CI: 3.93–16.66). Third-generation TKIs were associated with a significantly reduced risk of ≥3AE compared with first-generation TKIs (Gef) in the cases of Osi (OR = 0.63, 95% CI: 0.30–1.33) and Fur (OR = 0.58, 95% CI: 0.24–1.43), but no significant difference was observed in the case of Aum (OR = 1.03, 95% CI: 0.47–2.23). Ch combined with certain other treatments resulted in a significantly increased risk of ≥3AE in comparison with Ch only (Bev + Ch: OR = 1.26, 95% CI 0.55–2.88; Gef + Ch: OR = 1.28, 95% CI 0.57–2.89) and in comparison with Gef only (Gef + Ch: OR = 3.30, 95% CI 2.02–5.39). Finally, the safety profile of the first-generation TKI Erl was better than that of Gef on this measure (OR = 0.28, 95% CI: 0.09–93). However, there were no significant differences among second-generation TKIs or among third-generation TKIs.

**FIGURE 9:**
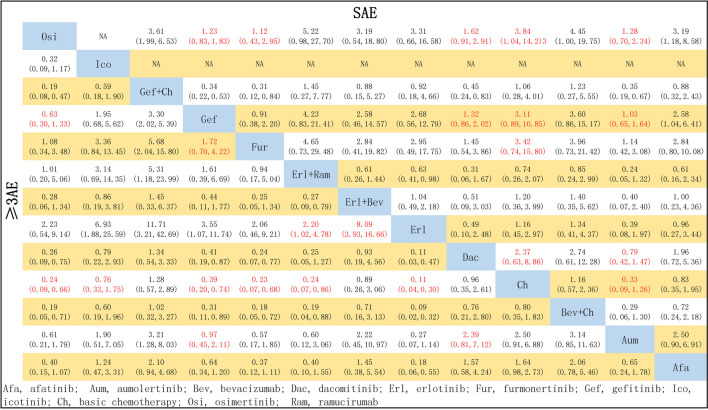
Pooled comparisons of treatments in patients with EGFR mutations, presented in the form of OR (95% CI) for the risk of incidence of ≥3 AE (lower triangle) and the risk of incidence of SAE (upper triangle). Each cell represents a comparison between the treatment specified on that row and the one specified in that column. An HR > 1 favors the therapy defined in the row. Important results are highlighted in red font. ≥ 3AE, adverse events of grade 3 or above; SAE, serious adverse events.

In terms of the risk of an SAE, compared with chemotherapy, the risk was significantly reduced by treatment with Osi (OR = 1.27, 95% CI: 0.59–2.72), Aum (OR = 1.09, 95% CI: 0.50–2.37), Fur (OR = 1.46, 95% CI: 0.60–3.68), Gef (OR = 1.09, 95% CI: 0.50–2.37), and Dac (OR = 1.46, 95% CI: 0.60–3.68). There were no significant differences between Osi, Aum, Fur, Gef, and Dac, or between Afa, Erl + Bev, Erl, Gef + Ch, Ch, Bev + Ch, and Erl + Ram (see Figure 9).

### 3.6 Subgroup analysis

A meta-analysis examining the effects of TKIs either combined with chemotherapy or without chemotherapy showed that treatment with TKIs combined with chemotherapy was associated with improved outcomes in terms of PFS (HR = 0.59, 95% CI: 0.50-0.71), but increased the risk of incidence of ≥3AE (OR = 3.30, 95%CI: 2.45–4.46). Similarly, a meta-analysis examining the effects of TKIs combined with anti-angiogenic agents or without anti-angiogenic agents indicated that treatment with TKIs combined with anti-angiogenic agents was associated with improved outcomes in terms of PFS (HR = 0.58, 95% CI: 0.88–0.70), but significantly increased the risk of incidence of ≥3AE (OR = 5.02, 95%CI: 1.8713.49). Detailed results are shown in Supplementary Data Sheet S2 (Supplementary Figures 5A–D).

### 3.7 Rank probabilities

**FIGURE 10:**
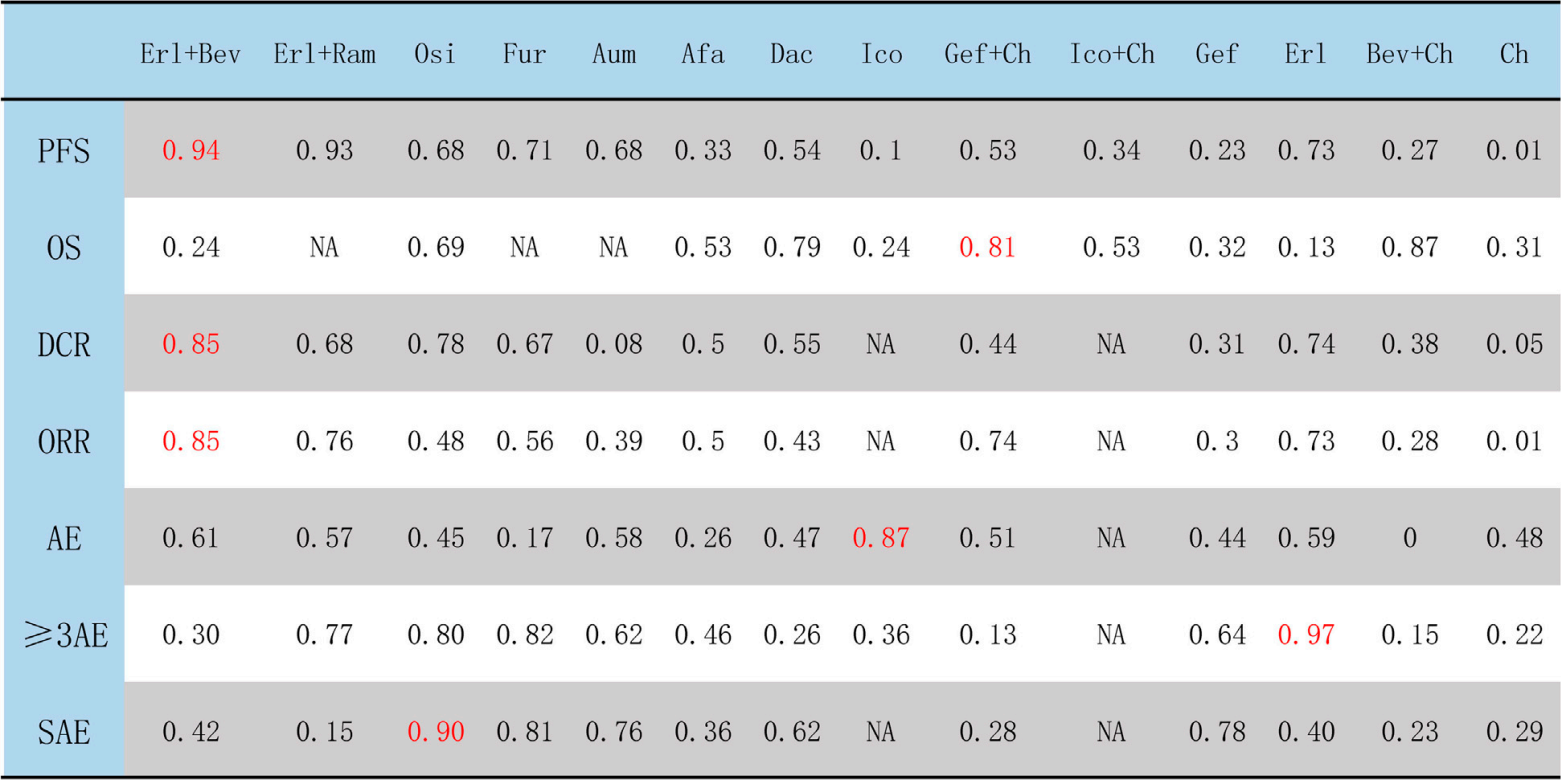
Ranking of treatments according to SUCRA. The higher the SUCRA score (which is always <1), the better the efficacy or safety of the treatment regimen as evaluated by the corresponding outcome measure. Afa, afatinib; Aum, aumolertinib; Bev, bevacizumab; Dac, dacomitinib; Erl, erlotinib; Fur, furmonertinib; Gef, gefitinib; Ico, icotinib; Ch, basic chemotherapy; Osi, osimertinib; Ram, ramucirumab; PFS, progression-free survival; OS, overall survival; DCR, disease control rate; ORR, objective response rate; AE, occurrence of any adverse events; ≥ 3AE, occurrence of any adverse events of grade 3 or above; SAE, occurrence of any serious adverse events.

**FIGURE 11:**
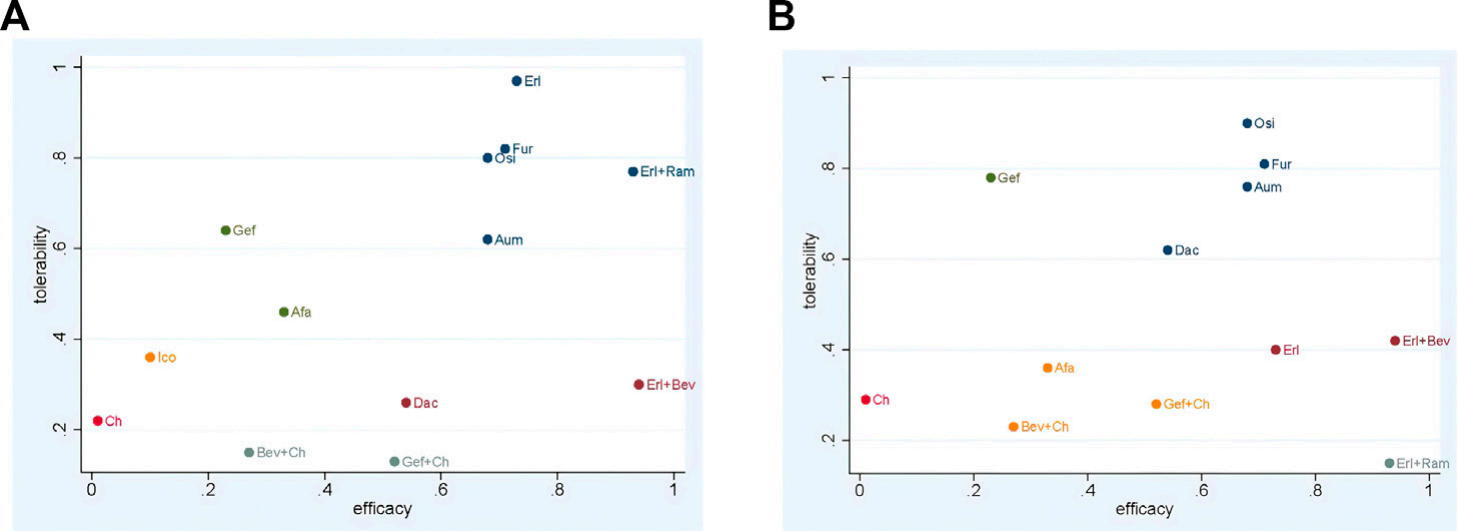
Network meta-analysis of comparisons between treatments in patients with advanced EGFR-mutated NSCLC **(A)** Evaluation of PFS combined with risk of AEs **(B)** Evaluation of PFS combined with risk of SAEs. Afa, afatinib; Aum, aumolertinib; Bev, bevacizumab; Dac, dacomitinib; Erl, erlotinib; Fur, furmonertinib; Gef, gefitinib; Ico, icotinib; Ch, basic chemotherapy; Osi, osimertinib; Ram, ramucirumab.

The ranking of treatments according to SUCRA evaluation is shown in Figure 10. Erl + Bev ranked first on PFS, DCR, and ORR, with SUCRA values of 0.94, 0.85, and 0.85, respectively; other associated SUCRA values were 0.24 (for OS), 0.30 (for >3AEs), and 0.42 (for SAE). Bev + Ch ranked first on OS, with a SUCRA value of 0.87; other associated SUCRA values were 0.27 (for PFS), 0.28 (for ORR), 0.15 (for ≥3AE), and 0.23 (for SAE). Ico ranked first on AE, with a SUCRA value of 0.87; other associated SUCRA values were 0.10 (for PFS), 0.24 (for OS), and 0.36 (for ≥3AE). Erl ranked first on ≥3AE, with a SUCRA value of 0.97; other associated SUCRA values were 0.73 (for PFS), 0.13 (for OS), 0.74 (for DCR), 0.73 (for ORR), 0.59 (for AE), and 0.40 (for SAE). Osi ranked first on SAE, with a SUCRA value of 0.90; other associated SUCRA values were 0.68 (for PFS), 0.69 (for OS), 0.78 (for DCR), 0.48 (for ORR), 0.45 (for AE), and 0.80 (for ≥3AE). According to the combined evaluation of PFS and incidence of ≥3AE, treatment regimens with good efficacy and safety were Osi, Aum, Fur, Erl, and Erl + Ram, as shown in Figure 11A. According to the combined evaluation of PFS and incidence of any SAE, Osi, Aum, Fur, and Dac also exhibited good efficacy and safety, as shown in Figure 11B.

## 4 Discussion

In this study, efforts were made to explore differences between treatment regimens (in particular, first-line treatments according to the 2023 guidelines and third-generation EGFR TKIs) for NSCLC patients harboring EGFR-sensitive mutations in the Asian population. To determine the best first-line regimen among the Asian population and explore the best first-line treatment for advanced EGFR-mutation NSCLC in this population, data from 19 RCTs were collated; these included studies of 14 first-line treatment regimens, and most were RCTs conducted in Asian countries, or in which at least 50% of the participants were Asian.

The results of the study showed that TKIs combined with angiogenesis inhibitors have relatively good efficacy in the treatment of advanced or metastatic NSCLC with EGFR mutation. In terms of PFS, the highest and second-highest SUCRA values were calculated for Erl + Bev and Erl + Ram, respectively, and this combination therapy demonstrated a certain degree of overall superiority Subgroup analysis indicated the efficacy of Erl combined with angiogenesis inhibitors: compared with Erl alone, the combination therapy showed significant improvement in terms of PFS, HR = 0.58 However, compared to Erl alone, the combination of angiogenesis inhibitors (Bev) with Erl failed to improve OS among patients with EGFR-sensitive mutations in advanced NSCLC; this finding was consistent with the results of a meta-analysis by Deng et al. (2022). Overall survival data for Erl + Ram were immature at the data cutoff time for this study. In comparison with Erl monotherapy, treatment with Erl + Ram or Erl + Bev was found to significantly prolong mPFS to more than 16 months (Seto et al., 2014; Nakagawa et al., 2019; Saito et al., 2019) in patients with EGFR mutation-positive NSCLC. However, the mechanism by which the combination of angiogenesis inhibitors with Erl improves survival compared to treatment with Erl alone remains unclear. Anti-angiogenic drugs can improve drug delivery (Ilhan-Mutlu et al., 2016), alter tumor blood vessels, and increase tumor uptake of drugs. It is also possible that angiogenesis inhibitors can inhibit the VEGF-A-mediated pathway to restore apoptosis (Pan et al., 2020). Dual blockade of the VEGF-A and EGFR pathways is a feasible first-line treatment strategy, and NSCLC resistance to EGFR mutations is still effective (Nagano et al., 2019). In the present meta-analysis, in terms of tumor load response, Erl + Bev was associated with the highest SUCRA values (i.e., the highest on DCR and ORR), and Erl + Ram was also associated with high values (ranking second on ORR and fourth on DCR), indicating the ultra-high sensitivity of this type of tumor to these combination therapies.

In terms of safety, the results suggested that combination treatments are associated with a high risk of adverse events. Regarding toxicity associated with anti-angiogenic drugs, adverse events were found to be more common among patients treated with angiogenic inhibitors combined with the Erl than among those treated with Erl monotherapy. In terms of SUCRA for the risk of ≥3AE, Erl + Bev ranked ninth, and for the risk of any SAE, Erl + Ram ranked lowest. The most commonly occurring adverse events of grade 3 or worse among patient groups treated with angiogenesis inhibitors combined with Erl were hypertension, rash, proteinuria, grade 4 neutropenia, and liver dysfunction or abnormal liver function. Hypertension, bleeding events (non-pulmonary bleeding), and proteinuria were significantly more common in the combination therapy groups than in the Erl monotherapy groups (Seto et al., 2014; Nakagawa et al., 2019; Saito et al., 2019; Yang et al., 2022). Overall, the severity of adverse reactions was found to be related not only to the drugs used in treatment but also to the patients’ pathological characteristics (ECOG score, metastasis). The failure of angiogenesis inhibitors combined with Erl to improve OS is probably related to the high risk of ≥3AE and/or SAE and the subsequent treatment required. Based on ongoing clinical trials (NCT03647592, NCT05507606, etc.), further efforts should also be made to investigate the role of combination therapy, including the use of anti-angiogenic drugs, in first-line treatment, and to investigate combination regimens with a low risk of adverse reactions, so as to explore potential combination therapy regimens that may be more suitable for Asian patients with EGFR-mutated metastatic NSCLC.

TKIs are the most important first-line treatment options. In terms of the choice between TKIs, the results presented here demonstrate that, compared with a first-generation TKI (Gef), second-generation TKIs (Afa and Dac) and third-generation TKIs (Osi, Aum, and Fur) present significant benefits, improving PFS and OS in the first-line treatment setting. SUCRA values for each TKI on PFS, in order, were as follows: Erl, 0.73; Fur, 0.71; Aum, 0.68; Osi, 0.68; Dac, 0.54; Afa, 0.33; Gef, 0.23; and Ico, 0.1. SUCRA values on OS were as follows: Dac, 0.79; Osi, 0.69; Afa, 0.53; and Gef, 0.32. In full agreement with the results of NMAs by Chen et al. (2022); Qi et al. (2022),; Yang et al. (2022), Erl ranked the highest, and single-agent TKI therapies with Erl and Ico probably outperformed chemotherapy only, although the network failed to form a closed ring In this study, all patient groups treated with chemotherapy were combined into a single chemotherapy group, and the use of different chemotherapy regimens might have led to inconsistent results. The FLAURA trial (NCT 02296125) (Soria et al., 2018; Ramalingam et al., 2020) indicated that Osi significantly prolongs mPFS (18.9 months) and mOS (38.6 months) compared with first-generation EGFR TKIs (Gef (n > 60%) or Erl, at 10.2 months and 31.8 months), while the APOLLO trial (NCT03849768) (Lu et al., 2022) indicated that Aum significantly prolongs mPFS (19.3 months) compared with first-generation EGFR TKIs (Gef: 9.9 months). Finally, in the FURLONG trial (NCT03787992) (Shi et al., 2022), Fur was found to significantly prolong mPFS (20.8 months) compared with first-generation EGFR TKIs (Gef: 10.1 months). In terms of safety, third-generation TKI treatments were confirmed to have a favorable safety profile: for SUCRA values on the incidence of ≥3 AE, Osi (0.80), Aum (0.62), and Fur (0.82) ranked below only Erl (0.97), and for the risk of incidence of any SAE, Osi (0.90), Aum (0.76), and Fur (0.81) ranked within the top four Generally, OS is considered to be the gold standard criterion for selection of the optimal treatment. The data on OS for Aum and Fur were not yet mature at the time of this study, but a trend could be observed suggesting that Fur offers superior benefits than Osi in terms of PFS and risk of incidence of ≥3 AE.

Third-generation TKIs are considered as either first-line or second-line treatments, based on which is the most beneficial. Clinicians typically consider third-generation TKIs as the first line and select the drug with the best chance of good performance, based on analysis of various measures, for second-line treatment after drug resistance. However, in reality, some patients may miss the opportunity to receive third-generation TKI treatment due to failure to detect the presence of the T790M mutation. Furthermore, not all patients can survive receiving second-line TKI treatment. The FLAURA trial showed that first-line Osi is similar to second-line Osi, still exerting a strongly beneficial effect (Ramalingam et al., 2020). Therefore, it is preferable to use third-generation TKIs as first-line therapies, but further trials are needed to provide more evidence to determine the most effective and reasonable line of treatment for the use of Osi. The investigation in clinical trials (NCT02856893 and NCT03790397, among others) of the optimal strategy in terms of sequencing of Gef and Osi in the first-line treatment of advanced NSCLC patients treated with EGFR TKIs will contribute to evaluation of the circumstances under which Osi can most beneficially be used as a first-line or second-line therapy. In addition, previous clinical data indicate that Osi achieves better penetration of the blood–brain barrier than Gef and thus benefits patients with brain metastases as a result of advanced lung cancer (Ballard et al., 2016; Goss et al., 2018). Based on the above evidence, it can reasonably be speculated that Osi/Aum/Fur may be the drugs of choice for advanced NSCLC after mutation.

Chemotherapy combined with other drugs is one of the essential first-line treatment options. In this study, evidence was found for significant prolongation of PFS and OS when TKIs are combined with chemotherapy, specifically in the cases of Gef/Ico + Ch. In terms of SUCRA values, Gef + Ch ranked eighth on PFS (0.53) and second on OS (0.81), while Ico + Ch ranked fourth on OS (0.53). The NEJ009 trial (Hosomi et al., 2020) demonstrated that, compared with Gef (11.2 months and 38.8 months), Gef + Ch is associated with significantly prolonged mPFS/mOS (20.9 months and 50.9 months, respectively); additionally, TKI combined with chemotherapy significantly improves survival rates in the case of advanced NSCLC with EGFR-activating mutations. Although Gef + Ch has been found to prolong PFS and OS, it is also associated with increased toxicity. In terms of SUCRA values, Gef + Ch ranked lowest on the incidence of ≥3AE (0.13), and the second-lowest ranking received by this treatment was for the risk of incidence of SAE (0.28). Combination chemotherapy regimens have been found to increase neutropenia, anemia, and thrombocytopenia, although the incidence of these toxicities is equal to or lower than those previously reported for carboplatin and pemetrexed (Cheng et al., 2016; Hosomi et al., 2020; Noronha et al., 2020; Yang et al., 2020). The present study indicated that, compared with Gef, Bev + Ch failed to significantly improve survival in cases of advanced NSCLC with EGFR mutations; no statistically significant difference between these treatments was observed.

The combination regimens examined here are strong candidates for prolonging the survival of patients in good condition. This study showed that first line use of third generation TKIs (especially Osi) prolonged PFS/OS compared to other single drug TKIs; Reduces the risk of adverse reactions compared with combination therapy. Given that third generation TKIs are effectiv e in treating NSCLC regardless of the presence or absence of T790M mutations, and that Gef and Erl have limited efficacy only for NSCLC with T790M mutations, first line administration of third generation TKIs (Osi/Aum/Fur) may be the best strategy. Ongoing clinical trials (NCT04035486 and NCT05507606, among others) have been investigating the survival benefits of third-generation TKIs (Osi) combined with chemotherapy or anti-angiogenic drugs, and the outcomes of these trials are urgently awaited to assess the potentially significant survival benefits and safety levels of these combinations for use in first-line treatment.

Despite these findings, there are certain limitations to the present study. First, heterogeneity was observed in the NMA, especially in subgroup analyses. Second, although this meta-analysis was based entirely on clinical trial data, the presence of confounding factors is still inevitable, leading to predictable publication and selection biases. Furthermore, unless individual patient data are evaluated, analyses are inherently subject to error, and most trials lacked long-term follow-up. To minimize the impact of follow-up issues, if multiple publications with different follow-ups were retrieved for the same trial, the publication reporting the most recent data was selected. Due to limited data, subgroup analyses were not performed for smoking status, sex, or ECOG score, which may have affected the final results. In further studies, meta-analyses should be conducted in these subgroups to explore the relative effects of treatment based on these clinical characteristics.

## 5 Conclusion

Based on the results of NMA and the approved indications of the combined treatment strategy, Fur, Osi, and Aum were potentially demonstrated in this review to be the best treatment for all patients in the Asian population with locally advanced or metastatic NSCLC with EGFR-positive mutations, under the combined considerations of survival benefits, tumor burden response, and safety. Erl + Bev was found to have the greatest efficacy in terms of PFS, although the risk of ≥3 AE or SAE was higher. In terms of DCR/ORR, Erl + Bev ranked highest; Erl ranked highest on the risk of incidence of ≥3 AE, and Osi ranked highest on the risk of SAE. Both Erl + Bev and Erl + Ram, or something else? showed beneficial effects in terms of PFS, DCR, and ORR, but further studies should still be carried out to establish ways to reduce the risk of adverse events. Nevertheless, this NMA provides potential evidence to enable clinicians to select the optimal treatment regimen for advanced NSCLC patients in the Asian population, and the most appropriate alternative regimen for patients who cannot tolerate the optimal regimen.

## Data Availability

The original contributions presented in the study are included in the article/Supplementary Material, further inquiries can be directed to the corresponding authors.
