# Supplementary material for: Comparison of the efficacy and safety of first-line treatments for of advanced EGFR mutation-positive non-small-cell lung cancer in Asian populations: a systematic review and network meta-analysis
Source: Front Pharmacol. 2023 Jul 6;14:1212313. doi: 10.3389/fphar.2023.1212313 (PMC10358853; doi:10.3389/fphar.2023.1212313)

Supplementary Material 3

Figure 1: Convergence diagram of PFS

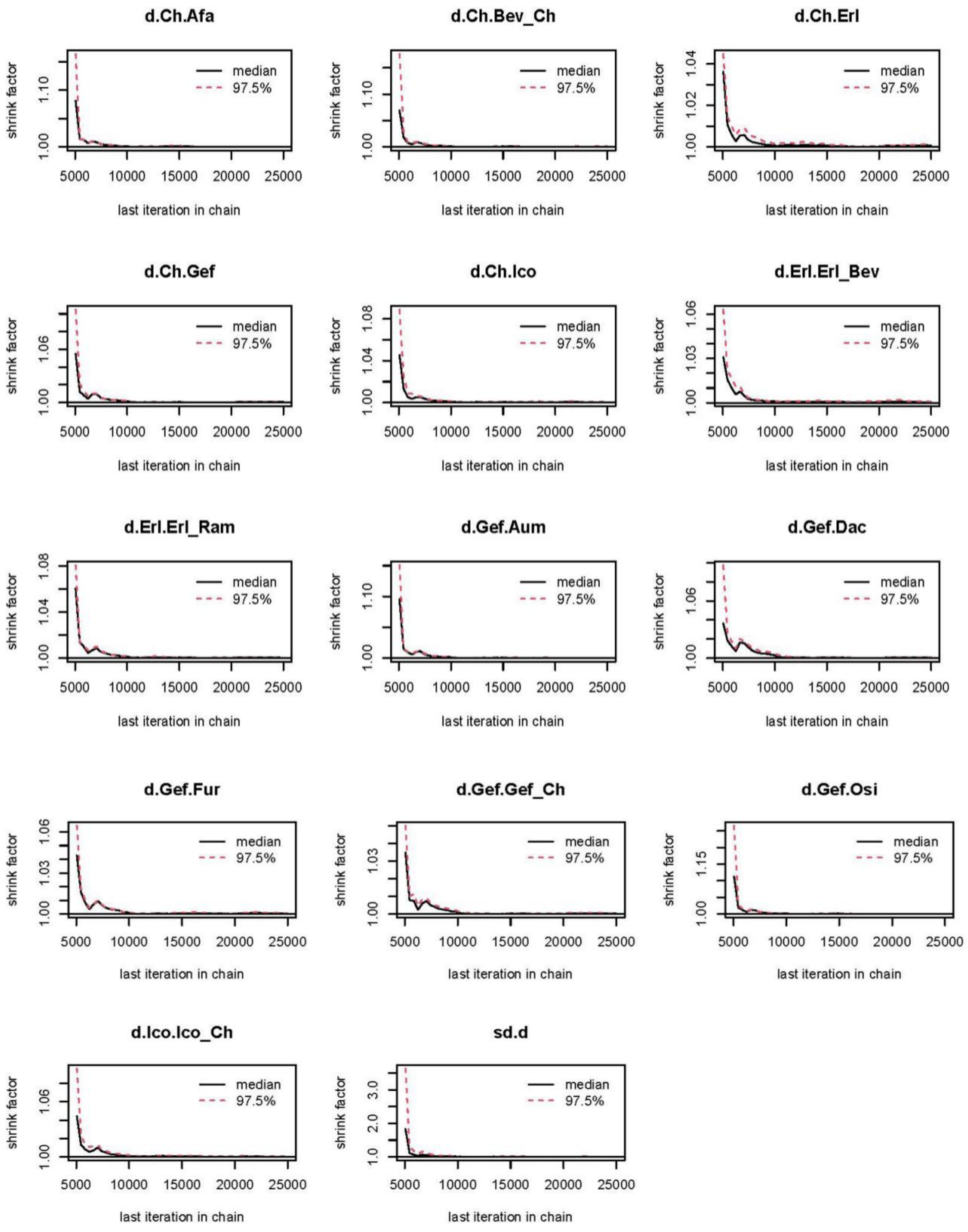

Figure 2: Convergence diagram of OS

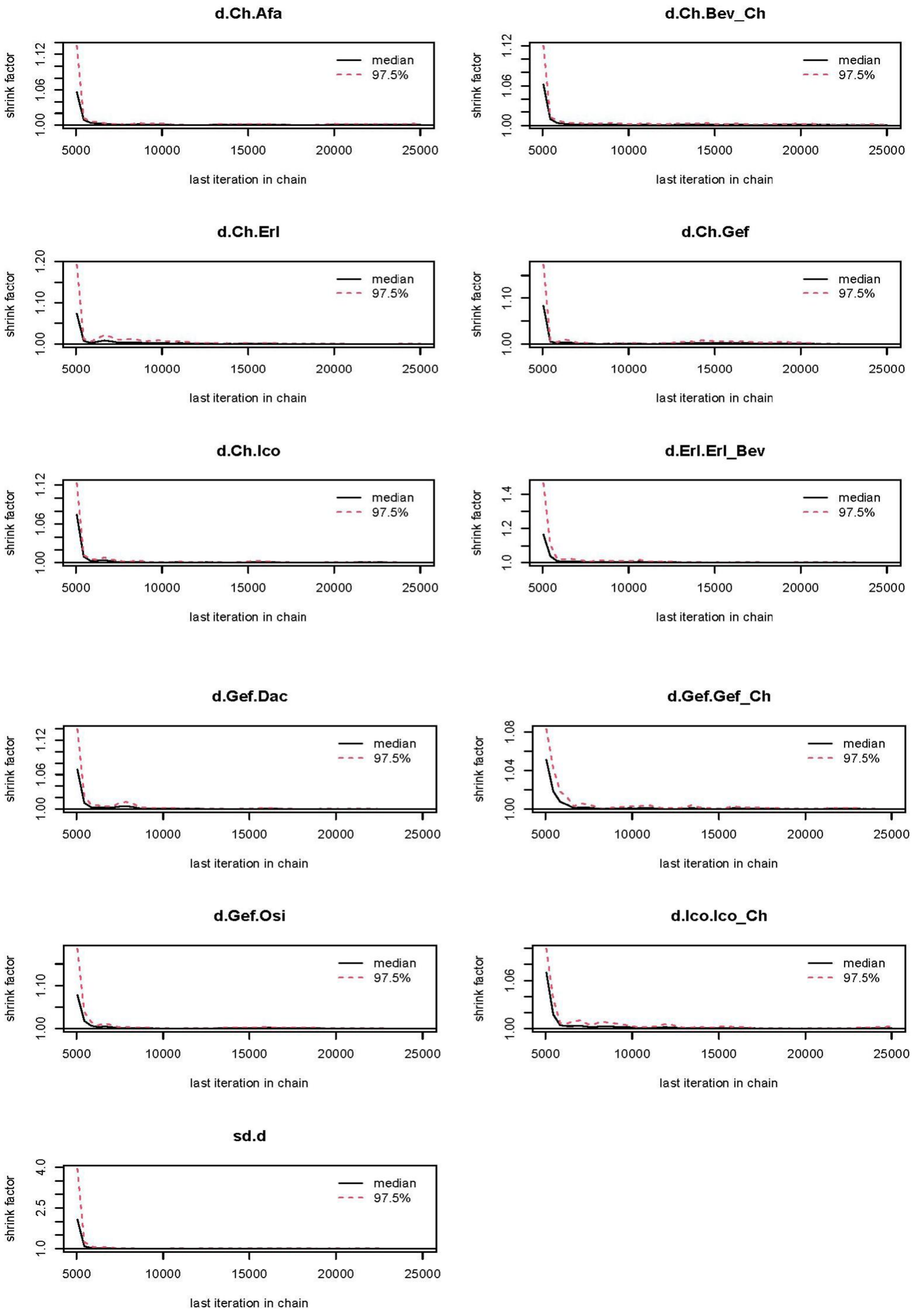

Figure 3:Funnel diagram of comparison outcomes in patients with advanced EGFR mutated NSCLC. (A) Funnel diagram for PFS. (B)Funnel diagram for OS. (C)Funnel diagram DCR. (D)Funnel diagram for ORR.(E)Funnel diagram for AE. (F)Funnel diagram for ≥3AE. (G)Funnel diagram plot for SAE.
(remarks Erl plus Bev: Erl+Bev/Erl_Bev, Erl plus Ram: Erl+Ram/Erl_Ram, Gef plus Ch: Gef+Ch/Gef_Ch, Erl plus Ch: Erl+Ch/Erl_Ch, Ico plus Ch: Ico+Ch/Ico_Ch)

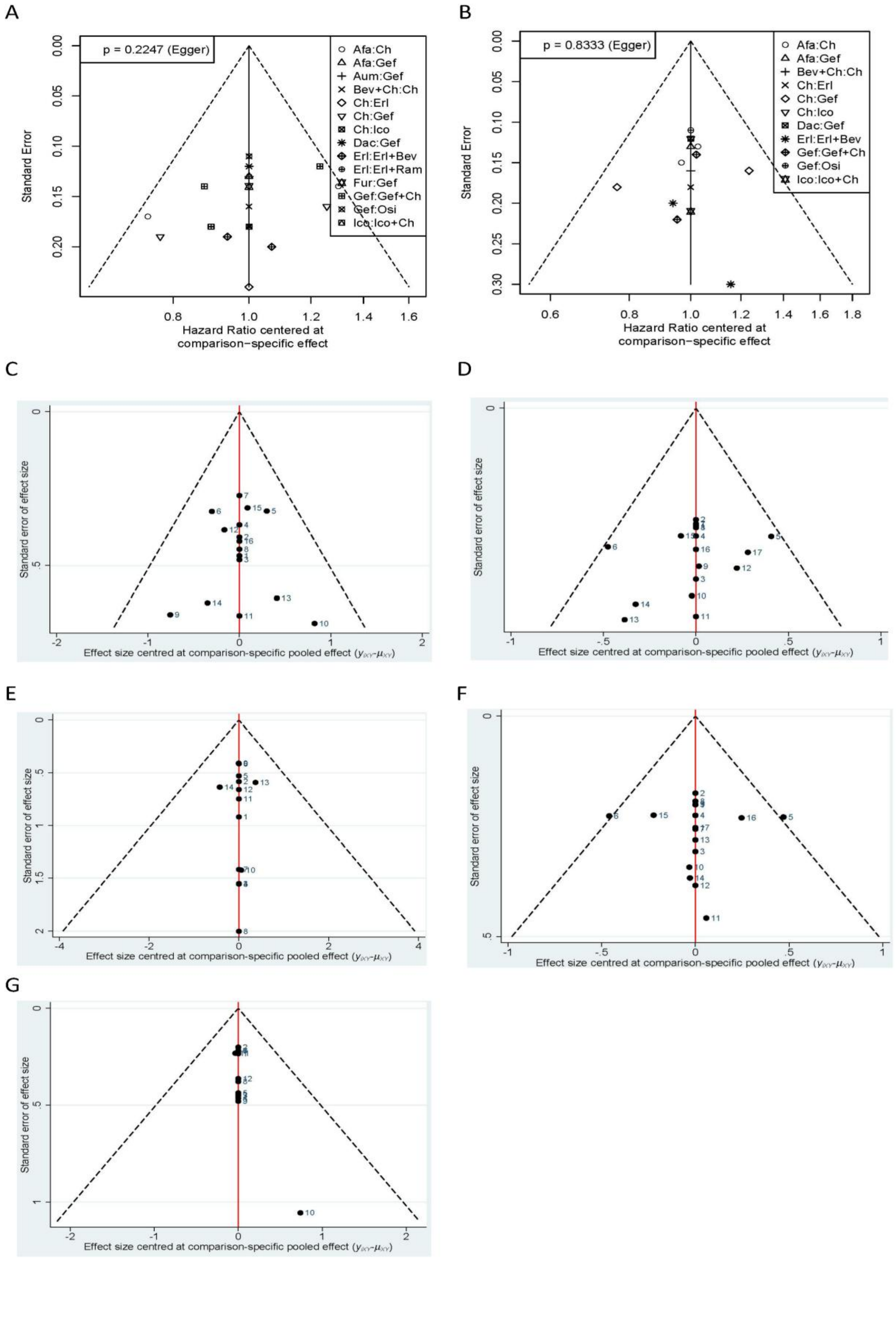

Figure 4: Contribution rates were combined for direct and indirect comparisons

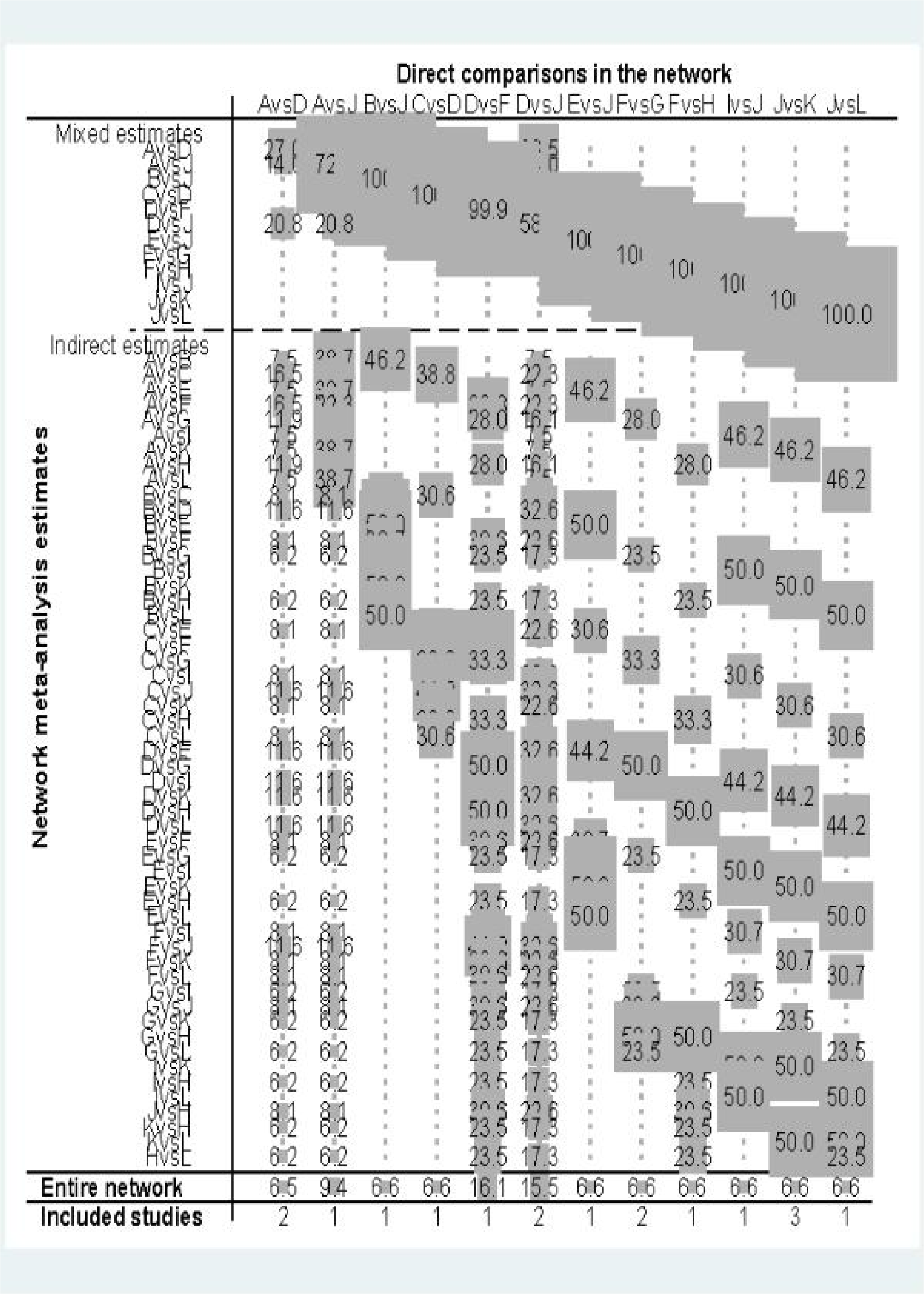

Figure 5:Subgroup analysis

(A) Meta-analysis of PFS of TKI with/without chemotherapy, (B)Meta-analysis of ≥3AE of TKI with/without chemotherapy, (C)Meta-analysis of PFS of TKI combined with/without antiangiogenic agents, (D)Meta-analysis of ≥3AE of TKI combined with/without antiangiogenic agents

A

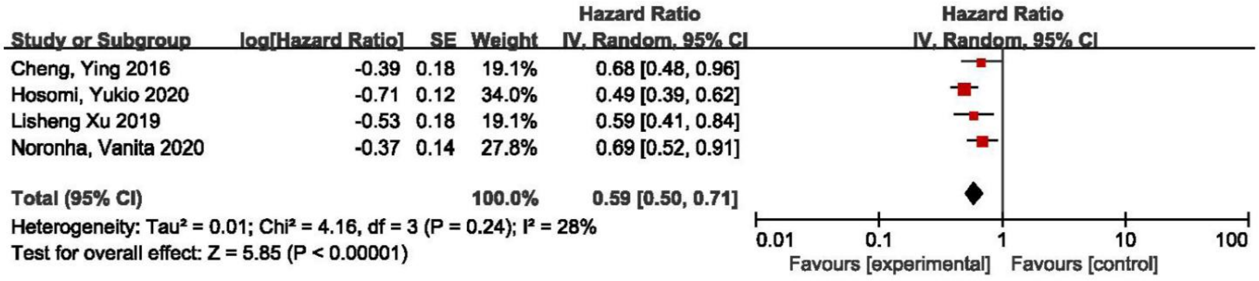

B

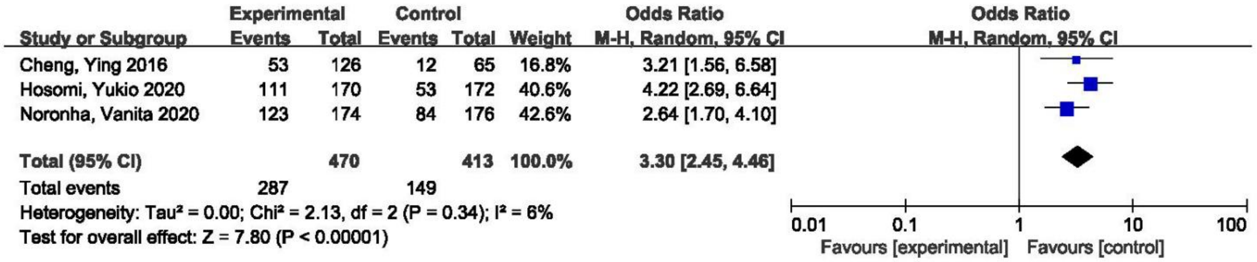

C

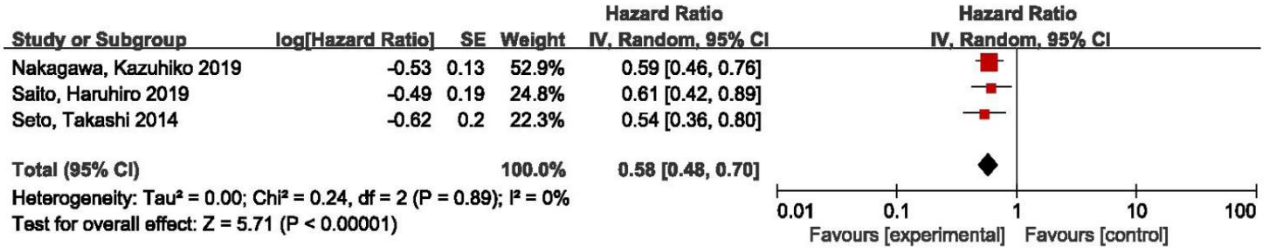

D

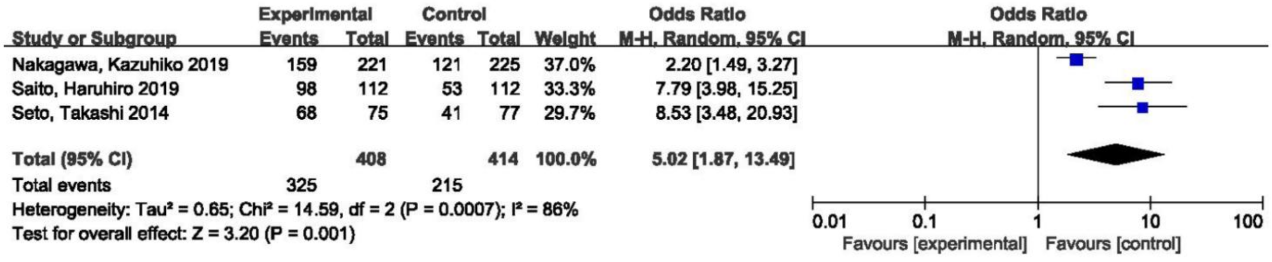

Supplement: Supplementary file 1 [file DataSheet2.PDF]
