# Supplementary material for: Comparison of the efficacy and safety of first-line treatments for of advanced EGFR mutation-positive non-small-cell lung cancer in Asian populations: a systematic review and network meta-analysis
Source: Front Pharmacol. 2023 Jul 6;14:1212313. doi: 10.3389/fphar.2023.1212313 (PMC10358853; doi:10.3389/fphar.2023.1212313)
Supplement: Supplementary file 2 [file DataSheet1.PDF]

Supplementary Material 1

pubmed Search Strategy

#1 (((((Gefitinib[MeSH Terms]) OR (N-(3-Chloro-4-fluorophenyl)-7-methoxy-6-(3-(4-morpholinyl)propoxy)-4-quinazolinamide[Title/Abstract])) OR (Iressa[Title/Abstract])) OR (ZD1839[Title/Abstract])) OR (ZD 1839[Title/Abstract])) AND (randomizedcontrolledtrial[Filter])((((Empagliflozin[Supplementary Concept]) OR (BI 10773[Title/Abstract])) OR (BI10773[Title/Abstract])) OR (BI-10773[Title/Abstract])) OR (Jardiance[Title/Abstract])

#2 ("erlotinib hydrochloride"[MeSH Terms] OR "hydrochloride erlotinib"[Title/Abstract] OR "erlotinib hcl"[Title/Abstract] OR "hcl erlotinib"[Title/Abstract] OR "OSI-774"[Title/Abstract] OR "OSI-774"[Title/Abstract] OR "OSI774"[Title/Abstract] OR "CP-358774"[Title/Abstract] OR ("358774"[All Fields] AND "CP"[Title/Abstract]) OR "cp 358 774"[Title/Abstract] OR ("358 774"[All Fields] AND "CP"[Title/Abstract]) OR "cp 358 774"[Title/Abstract] OR ("CP358"[All Fields] AND "774"[Title/Abstract]) OR "CP-358774"[Title/Abstract] OR "CP358774"[Title/Abstract] OR "11C-erlotinib"[Title/Abstract] OR "11C-erlotinib"[Title/Abstract] OR "Erlotinib"[Title/Abstract] OR (((("n"[All Fields] AND "3-ethynylphenyl"[All Fields]) AND "6 7 bis"[All Fields]) AND "2-methoxyethoxy"[All Fields]) AND "quinazolin-4-amine"[Title/Abstract]) OR "Tarceva"[Title/Abstract]) AND (randomizedcontrolledtrial[Filter])

#3 (icotinib[MeSH Terms]) OR (4-((3-ethynylphenyl)amino)-6,7-benzo-12-crown-4-quinazoline[Title/Abstract]) Filters: Randomized Controlled Trial

#4 (((((((((((Afatinib[MeSH Terms]) OR ((2E)-N-(4-(3-Chloro-4-fluoroanilino)-7-(((3S)-oxolan-3-yl)oxy)quinoxazolin-6-yl)-4-(dimethylamino)but-2-enamide[MeSH Terms])) OR (BIBW-2992-MA2[MeSH Terms])) OR (BIBW 2992 MA2[MeSH Terms])) OR (BIBW-2992MA2[MeSH Terms])) OR (BIBW 2992MA2[MeSH Terms])) OR (BIBW2992 MA2[MeSH Terms])) OR (Afatinib Maleate[MeSH Terms])) OR (BIBW 2992[MeSH Terms])) OR (BIBW2992[MeSH Terms])) OR (BIBW-2992[MeSH Terms])) OR (Gilotrif[MeSH Terms])) OR (Afatinib Dimaleate[MeSH Terms]) Filters: Randomized Controlled Trial

#5 (((((Dacomitinib[MeSH Terms]) OR (Vizimpro[Title/Abstract])) OR (N-(4-(3-chloro-4-fluoroanilino)-7-methoxy-6-quinazolinyl)-4-(1-piperidinyl)-2-butenamide[Title/Abstract])) OR (PF 00299804[Title/Abstract])) OR (PF00299804[Title/Abstract])) OR (PF-00299804[Title/Abstract]) Filters: Randomized Controlled Trial

#6 (((((((((((osimertinib[MeSH Terms]) OR (N-(2-((2-(dimethylamino)ethyl)methylamino)-4-methoxy-5-((4-(1-methyl-1H-indol-3-yl)-2-pyrimidinyl)amino)phenyl)-2-propenamide[Title/Abstract])) OR (mereletinib[Title/Abstract])) OR (osimertinib mesylate[Title/Abstract])) OR (osimertinib mesilate[Title/Abstract])) OR (mereletinib mesilate[Title/Abstract])) OR (N-(2-((2-(dimethylamino[Title/Abstract])ethyl)methylamino)-4-methoxy-5-((4-(1-methyl-1H-indol-3-yl)-2-pyrimidinyl)amino)phenyl)-2-propenamide

methanesulfonate (1:1))) OR (AZD9291 mesylate[Title/Abstract])) OR (mereletinib mesylate[Title/Abstract])) OR (AZD-9291 mesylate[Title/Abstract])) OR (AZD9291[Title/Abstract])) OR (AZD-9291[Title/Abstract])) OR (Tagrisso[Title/Abstract]) Filters: Randomized Controlled Trial

#7 (((Aumolertinib[MeSH Terms]) OR (almonertinib[Title/Abstract])) OR (N-(5-((4-(1-cyclopropylindol-3-yl)pyrimidin-2-yl)amino)-2-(2-(dimethylamino)ethyl-methylamino)-4-methoxyphenyl)prop-2-enamide[Title/Abstract])) OR (HS-10296[Title/Abstract]) Filters: Randomized Controlled Trial

#8 (((((Furmonertinib[MeSH Terms]) OR (N-(2-(2-(dimethylamino)ethyl-methylamino)-5-((4-(1-methylindol-3-yl)pyrimidin-2-yl)amino)-6-(2,2,2-trifluoroethoxy)pyridin-3-yl)prop-2-enamide[Title/Abstract])) OR (AST2818[Title/Abstract])) OR (furmonertinib mesylate[Title/Abstract])) OR (furmonertinib[Title/Abstract])) OR (Ivesa[Title/Abstract]) Filters: Randomized Controlled Trial

#9 ((((Bevacizumab[MeSH Terms]) OR (Mvasi[Title/Abstract])) OR (Bevacizumab-awwb[Title/Abstract])) OR (Bevacizumab awwb[Title/Abstract])) OR (Avastin[Title/Abstract]) Filters: Randomized Controlled Trial

#10 ((((((Ramucirumab[MeSH Terms]) OR (LY3009806[Title/Abstract])) OR (Cyramza[Title/Abstract])) OR (IMC 1121B[Title/Abstract])) OR (IMC1121B[Title/Abstract])) OR (IMC-1121B[Title/Abstract])) OR (1121B[Title/Abstract]) Filters: Randomized Controlled Trial

#11 (((((((((((Non-Small Cell Lung Cancer[MeSH Terms]) OR (Carcinoma, Non Small Cell Lung[Title/Abstract])) OR (Carcinomas, Non-Small-Cell Lung[Title/Abstract])) OR (Lung Carcinoma, Non-Small-Cell[Title/Abstract])) OR (Lung Carcinomas, Non-Small-Cell[Title/Abstract])) OR (Non-Small-Cell Lung Carcinomas[Title/Abstract])) OR (Non-Small-Cell Lung Carcinoma[Title/Abstract])) OR (Non Small Cell Lung Carcinoma[Title/Abstract])) OR (Carcinoma, Non-Small Cell Lung[Title/Abstract])) OR (Non-Small Cell Lung Carcinoma[Title/Abstract])) OR (Non-Small Cell Lung Cancer[Title/Abstract])) OR (Nonsmall Cell Lung Cancer[Title/Abstract]) Filters: Randomized Controlled Trial

#12 #1 or #2 or #3 or #4 or #5 or #6 or #7 or #8 or #9 or #10 and #11

Embase Search Strategy

#1 'non-small cell lung cancer'/exp

#2 'carcinoma, non small cell lung':ti,ab OR 'carcinomas, non-small-cell lung':ti,ab OR 'lung carcinoma, non-small-cell':ti,ab OR 'lung carcinomas, non-small-cell':ti,ab OR 'non-small-cell lung carcinomas':ti,ab OR 'non-small-cell lung carcinoma':ti,ab OR 'non small cell lung carcinoma':ti,ab OR 'carcinoma,non-small cell lung':ti,ab OR 'non-small cell lung carcinoma':ti,ab OR 'non-small cell lung cancer':ti,ab OR 'nonsmall cell lung cancer':ti,ab

#3 #1 OR #2
#4 'tyrosine protein kinase inhibitors'/exp
#5 'tyrosine kinase inhibitors':ti,ab OR 'inhibitors, tyrosine kinase':ti,ab OR 'kinase inhibitors, tyrosine':ti,ab OR 'tki tyrosine kinase inhibitors':ti,ab OR 'tyrosine kinase inhibitor':ti,ab
#6 #4 OR #5
#7 'bevacizumab'/exp
#8 'mvasi':ti,ab OR 'bevacizumab-awwb':ti,ab OR 'bevacizumab awwb':ti,ab OR 'avastin':ti,ab
#9 #7 OR #8
#10 'ramucirumab'/exp
#11 'ly3009806':ti,ab OR 'cyramza':ti,ab OR 'imc 1121b':ti,ab OR 'imc1121b':ti,ab OR 'imc-1121b':ti,ab OR '1121b':ti,ab
#12 #10 OR #11
#13 #6 OR #9 OR #12
#14 'randomized controlled trial'/exp
#15 'randomized':ti,ab OR 'placebo':ti,ab
#16 #14 OR #15
#17 #3 AND #13 AND #16

Cochrane Search Strategy

#1 MeSH descriptor:[Carcinoma, Non Small Cell Lung] explode all trees
#2 (Non-Small Cell Lung Cancer:ti,ab,kw) or (Carcinomas, Non-Small-Cell Lung:ti,ab,kw) or (Lung Carcinoma, Non-Small-Cell:ti,ab,kw) or (Lung Carcinomas, Non-Small-Cell:ti,ab,kw) or (Non-Small-Cell Lung Carcinomas:ti,ab,kw) or (Non-Small-Cell Lung Carcinoma:ti,ab,kw) or (Non Small Cell Lung Carcinoma:ti,ab,kw) or (Carcinoma, Non-Small Cell Lung:ti,ab,kw) or (Non-Small Cell Lung Carcinoma:ti,ab,kw) or (Non-Small Cell Lung Cancer:ti,ab,kw) or (Nonsmall Cell Lung Cancer:ti,ab,kw)
#3 #1 or #2
#4 MeSH descriptor:[Tyrosine Protein Kinase Inhibitors] explode all trees
#5 (Tyrosine Kinase Inhibitors:ti,ab,kw) or (Inhibitors, Tyrosine Kinase:ti,ab,kw) or (Kinase Inhibitors, Tyrosine:ti,ab,kw) or (TKI Tyrosine Kinase Inhibitors:ti,ab,kw) or (Tyrosine Kinase Inhibitor:ti,ab,kw)
#6 #4 or #5
#7 MeSH descriptor:[Bevacizumab] explode all trees
#8 (Mvasi:ti,ab,kw) or (Bevacizumab-awwb:ti,ab,kw) or (Bevacizumab awwb:ti,ab,kw) or (Avastin:ti,ab,kw)
#9 #7 or #8
#10 MeSH descriptor:[Ramucirumab] explode all trees
#11 (LY3009806:ti,ab,kw) or (Cyramza:ti,ab,kw) or (IMC 1121B:ti,ab,kw) or (IMC1121B:ti,ab,kw) or (IMC-1121B:ti,ab,kw) or (1121B:ti,ab,kw)
#12 #10 or #11
#13 #6 or #9 or #12
#14 #3 and #13
